# Do Chameleons Lead Better? A Meta-Analysis of the Self-Monitoring and Leadership Relationship

**DOI:** 10.1177/01461672231210778

**Published:** 2023-11-25

**Authors:** Linghe Lei, Chen Wang, Jonathan Pinto

**Affiliations:** 1 University of Reading, UK; 2 Western Michigan University, Kalamazoo, USA; 3 Imperial College London, UK

**Keywords:** self-monitoring, leadership, personality, meta-analysis

## Abstract

The relationship between self-monitoring and leadership has been debated. We attempt to resolve this debate through a meta-analysis (*N* = 9,029 across 55 samples). Since this is the first meta-analysis that focuses on this relationship, we were able to study both focal constructs at a granular level. As hypothesized, self-monitoring is positively associated with *leadership emergence* and *leadership effectiveness*. Whereas self-monitoring is positively related to *managerial leadership*, its relationship with *transactional leadership* is non-significant. Contrary to our prediction that self-monitoring is negatively related to *authentic leadership* and to *transformational leadership*, we found positive relationships. Importantly, the relationship between self-monitoring and leadership variables is typically non-significant when the latter is measured by subordinate ratings. This casts doubt on the general finding that self-monitoring is positively related to leadership. Also, the relationships significantly differ when self-monitoring was measured by different scales. Implications for theory and practice are discussed.

Since Snyder (1974) introduced the *self-monitoring* construct to describe individual differences in regulating self-expressive behaviors in response to situational and social cues, it has generated substantial academic scholarship. As Bedeian and Day (2004, p. 705) assert: “There is little debate that self-monitoring is one of the most heavily researched personality constructs in social and applied psychology.” Self-monitoring of one’s self-presentation to meet situational demands is referred to as being chameleon-like (Snyder, 1979), and individuals who score higher on self-monitoring scales, called *high self-monitors* (HSMs) are referred to as chameleons (e.g., Bizzi & Soda, 2011; Rowatt et al., 1998). In contrast, those who score lower are *low self-monitors* (LSMs) and are described as principled persons (Snyder, 1979).

Although chameleons are individuals that people do not tend to hold in high esteem as compared to principled individuals, research finds that self-monitoring is associated with several positive work outcomes (Kudret et al., 2019). For example, self-monitoring is positively associated with getting recruited (Higgins & Judge, 2004), job performance (e.g., Wang et al., 2015), and career progression (Kilduff & Day, 1994). Even though some of these dependent variables should be associated with leadership, the relationship between self-monitoring and leadership is not clear, and this is one of the puzzles that has motivated this study.

Research on the relationship between self-monitoring and leadership variables has produced mixed findings. For instance, although several studies found a positive relationship between self-monitoring and *leadership emergence* (e.g., Eby et al., 2003; Ellis, 1988), others found non-significant relationships (e.g., Côté et al., 2010). Whereas some studies found a positive relationship between self-monitoring and *leadership effectiveness* (e.g., Anderson & McLenigan, 1987; Campbell, 1993), others found the relationship to be either non-significant (e.g., Douglas & Ammeter, 2004) or negative (e.g., Becker, 2003). Research findings are also mixed regarding the relationship between self-monitoring and leadership styles. For instance, although Chaudhary and Panda (2019) found a positive relationship between self-monitoring and *authentic leadership*, Tate (2008) found a non-significant relationship. Similarly, Jung and Sosik (2006) found a positive relationship between self-monitoring and *transformational leadership*, but Sosik et al. (2002) found the relationship to be non-significant.

The confusing relationship between self-monitoring and leadership has also been debated in the literature by Bedeian and Day (Bedeian & Day, 2004). Bedeian states: “*What puzzles me is that the description of typical high self-monitors, as reported in the literature, does not match what either my personal experiences or decades of research in other areas indicate is the portfolio of a* ***real leader***” (Bedeian & Day, 2004, p. 688) [emphasis added]. In response, Day states: “*why would other people allow high self-monitors* ***to emerge as leaders****? A core reason for this is that they like and identify with high self-monitors because they do a better job of meeting* ***interpersonal expectations***” (Bedeian & Day, 2004, p. 699) [emphasis added]. Day’s standpoint focuses on *leadership emergence*, which is not the same as *leadership effectiveness* or being a real leader. Bedeian posits that HSMs’ chameleon-like nature does not align with the characteristics of a “real leader.” Real leaders strive for authenticity and exhibit a low motivation to self-monitor. But Day’s response emphasizes HSMs’ superior interpersonal abilities. Thus, Bedeian and Day based their arguments on two different components of self-monitoring, namely motivation and ability, respectively.

Given the mixed findings and the multifaceted impact of self-monitoring on leadership, our research seeks to discern which leadership phenomena and measures are either enhanced or hindered by self-monitoring. We thus applied a meta-analysis to investigate the relationship between self-monitoring and six leadership variables: leadership emergence, leadership effectiveness, authentic leadership, transformational leadership, transactional leadership, and managerial leadership (variables selected subject to data availability). Meta-analysis is a proper method because it draws productive lines of research by providing a summary of prior findings and identifies issues to spark new research (Lord et al., 2017).

Our meta-analysis, comprising 9,029 individuals across 55 samples, is the first meta-analysis that exclusively concentrates on the relationship between self-monitoring and leadership. Previous meta-analyses have only partially examined the self-monitoring leadership relationship. Day et al. (2002) examined the relationship between self-monitoring and “leadership” (where the authors incorporated both leadership emergence and leadership behaviors in this category) among a host of other dependent variables. Moreover, this study was conducted two decades ago. Another meta-analysis by Hoffman et al. (2011) has only examined the relationship between self-monitoring (among numerous individual attributes) and leadership effectiveness. Our focused approach allows an in-depth investigation of the relationships, including two moderators from the independent variable and the dependent variable, respectively: (a) the scale used to measure self-monitoring and (b) the source of leadership ratings (e.g., subordinates, peers, or supervisors). The granular examination of these relationships not only contributes to a clearer understanding of the impact of self-monitoring on leadership but also adds to a finer-grained understanding of both constructs.

## Literature Review

### Self-Monitoring

Self-monitoring refers to “differences in the extent to which people value, create, cultivate, and project social images and public appearances” (Gangestad & Snyder, 2000, p. 531). This definition suggests that self-monitoring encompasses two aspects—one, the extent to which people value social images and public impressions, and two, the extent to which they are able to cultivate an appropriate social image in public appearances. The first is the motivational aspect of self-monitoring, and the second is the ability to self-monitor. These two aspects are clearly delineated in a recent definition of self-monitoring (Kudret et al., 2019, p. 194): “*Self-monitoring captures one’s* ***willingness*** *and* ***adeptness*** *at modifying their social images in line with situational demands, and behaving in line with social role expectations of others*” [emphasis added]. The validity of these two dimensions is further reinforced by Warech et al. (1998), who have developed scales to measure self-monitoring motivation and ability separately.

The motivation and ability dimensions are also pertinent in distinguishing between HSMs and LSMs. HSMs are high on both dimensions, that is, those who are “***willing and able*** *to project images designed to impress others*” [emphasis added] (Gangestad & Snyder, 2000, p. 531), whereas LSMs “*lack* ***either the ability or the motivation*** *to so regulate their expressive self-presentation*” [emphasis added] (Snyder & Gangestad, 1986, p. 125). Though some research defines LSMs as lacking both motivation and ability to self-monitor (e.g., Gangestad & Snyder, 2000), we follow Snyder and Gangestad’s (1986) seminal work and several subsequent research (e.g., Allen et al., 2005; Roberson & Williamson, 2012) and adopt the definition that LSMs lack either the motivation or ability to self-monitor. That is, since encompassing *both* motivation and ability leads to self-monitoring (thereby being HSMs), those who lack *either* the motivation or the ability would *not* result in high self-monitoring (thus categorized as LSMs).

#### Self-Monitoring Motivation

Research has examined the different dimensions of self-monitoring motivation, encompassing protective motivation and acquisitive motivation (Lennox, 1988). Protective motivation refers to passively conforming to fit into a situation to avoid rejection, associated with the “getting along” aspect of social life according to socioanalytic theory (Day & Schleicher, 2006; Hogan et al., 1985). Acquisitive motivation refers to actively engaging in social settings to cultivate a positive impression, linked to the “getting ahead” aspect as per socioanalytic theory (Day & Schleicher, 2006; Gangestad & Snyder, 2000).

Despite acknowledging that self-monitoring concerns both protective and acquisitive motivations, research indicates that the ultimate motivation of self-monitoring is the acquisitive motive (Day & Schleicher, 2006; Gangestad & Snyder, 2000). Engaging in a protective motivation, such as striving for acceptance and approval, contributes to the acquisitive motive to get ahead (Day & Schleicher, 2006). According to Gangestad and Snyder (2000, p. 547), the primary question regarding HSMs’ motivations is: “What do high self-monitors strive to acquire?” Based on a review of empirical studies, they elucidated that the ultimate acquisitive goal of self-monitoring is status enhancement (Gangestad & Snyder, 2000). This conclusion is further supported by Fuglestad and Snyder’s (2010) review and by Wilmot and Ones (2018), who found a positive meta-analytic relationship between self-monitoring and status motivations.

Self-monitoring motivation can have both positive and negative effects on leadership. On one hand, it enables an individual to navigate social settings effectively, facilitating leadership processes (Judge et al., 2009). On the other hand, this motivation can be self-serving and lead to instrumental behaviors, potentially undermining leadership processes (Sosik et al., 2002).

#### Self-Monitoring Ability

According to Snyder (1974), self-monitoring ability includes “an acute sensitivity to the cues in a situation which indicate what expression or self-presentation is appropriate” (p. 527) and “the ability to control and modify one’s self-presentation and expressive behavior” (p. 529). These two abilities have been specifically defined as two subdimensions of self-monitoring in Lennox and Wolfe’s (1984, p. 1361) self-monitoring definition, namely “sensitivity to expressive behavior of others” and “ability to modify self-presentation.”

Self-monitoring ability is widely recognized in the literature. Many scholars even refer to the self-monitoring construct as “self-monitoring ability” (e.g., Becker et al., 2002) or “self-monitoring skill” (e.g., Child & Agyeman-Budu, 2010), rather than considering it as a holistic individual attribute, even though Gangestad and Snyder (2000) underscored the motivational element of the self-monitoring construct. Overall, self-monitoring abilities, regarded as key dimensions of the self-monitoring construct, contribute positively to leadership. Sensitivity is a key ability in leadership (Keller, 1999), and the ability to modify self-presentation is critical for communication skills (Duran & Spitzberg, 1995), which also play a significant role in leadership.

#### Self-Monitoring Measurement Scales

All the studies included in this meta-analysis used one of the three most-acknowledged self-monitoring scales, that is, the original self-monitoring scale, SMS (Snyder, 1974), the revised self-monitoring scale, RSMS (Lennox & Wolfe, 1984), and the shorter version of the original self-monitoring scale, SMS-Reduced, or SMS-R (Snyder & Gangestad, 1986). Whereas the SMS and SMS-R operationalize self-monitoring as including both motivation and ability (Gangestad & Snyder, 2000), the RSMS is exclusively focused on self-monitoring ability (Lennox & Wolfe, 1984; O’Cass, 2000), which include two subscales, that is, “sensitivity to expressive behavior of others” and “ability to modify self-presentation” (Lennox & Wolfe, 1984, p. 1361).

### Leadership

Leadership is a prominent concern in all types of organizations (Day et al., 2014) and is one of the most important issues in human sciences and applied psychology (Gardner et al., 2020). The influencing or persuading aspect is central to leadership. Antonakis and Day (2017, p. 5) define leadership as “a formal or informal contextually rooted and goal-influencing process that occurs between a leader and a follower, groups of followers, or institutions.” Hogan et al.’s (1994, p. 493) definition is more specific: “Leadership involves persuading other people to set aside for a period of time their individual concerns and to pursue a common goal that is important for the responsibilities and welfare of a group.” Influence is also related to self-monitoring because high self-monitoring could be interpreted as fulfilling a desire to influence (Gangestad & Snyder, 2000).

In terms of studying leadership in general, researchers have parsed it into two broad categories, that is, leadership emergence and leadership effectiveness (Judge et al., 2002). This categorization is important because not all emergent leaders are good leaders (Hogan et al., 1994). Scholarship on leadership has also generated a plethora of specific theories and constructs (Dinh et al., 2014). Early theories include trait theories, behavioral theories, contingency theories, and implicit leadership theories. More recent leadership constructs and theories include authentic leadership, ethical leadership, servant leadership, strategic leadership, team leadership, and transformational leadership (Gardner et al., 2020). Not all these constructs have been examined in relation to self-monitoring. Thus, based on data availability, we examined the relationship between self-monitoring and the following six leadership variables: leadership emergence, leadership effectiveness, authentic leadership, transformational leadership, transactional leadership, and managerial leadership.

## Hypothesis Development

### Leadership Emergence

Leadership emergence (Judge et al., 2002) is one of three similar constructs; the other two are *emergent leadership* and *leader emergence*. The three concepts are usually used interchangeably (Badura et al., 2022). In this paper, we use the term “leadership emergence.” Leadership emergence refers to the extent to which an individual is viewed as a leader by others “who typically have only limited information about that individual’s performance” (Judge et al., 2002, p. 767). Leadership emergence involves an individual coming to be seen as influential and becoming a leader in a team, organization, or among others (Badura et al., 2022; Judge et al., 2002). It could occur in formal or informal leadership roles, including the implicit and explicit granting of the leadership role by others (Badura et al., 2022).

We hypothesize a positive relationship between self-monitoring and leadership emergence for the following four reasons. First, leadership emergence is contingent upon the subjective perception of leadership by others (Badura et al., 2022). This perception is rooted in *implicit leadership theory* (Lord et al., 1984), which posits that emergent leaders are determined based on others’ perceptions of what a leader should be like, regardless of other key factors such as the situation, the team, or the task (Lord et al., 2020). According to implicit leadership theory, attributes of dominance and extraversion are crucial in shaping leader perceptions, and thus individuals manifesting these traits are often seen as leader-like and emerge as leaders (Hogan et al., 1994; Judge et al., 2002). Compared to LSMs, HSMs are more dominant in social interactions, usually becoming the center of attention and initiating conversations (Foti & Hauenstein, 2007; Ickes & Barnes, 1977). In addition, HSMs display more extraverted and expressive behaviors than LSMs to actively display socially desirable behaviors. Research also indicates that the self-monitoring construct encompasses the extraversion factor (Parks-Leduc et al., 2014) and public performance (Briggs & Cheek, 1988), reinforcing the pronounced extraversion aspect of self-monitoring. Thus, given the higher level of dominance and extraversion displayed by HSMs, they are more likely to emerge as leaders compared to LSMs.

Second, in comparison to LSMs, HSMs exhibit higher levels of attentiveness and responsiveness to the expectations of group members and are more adaptive to changing situations (Snyder, 1979). When collaborating within groups, HSMs tend to display greater flexibility and demonstrate superior interpersonal capabilities (Hall et al., 1998). These interpersonal capabilities align with the leader prototype per implicit leadership theory (Lord et al., 1984), making HSMs more likely to be perceived as leaders.

Third, socioanlaytic theory (Hogan et al., 1985) posits that people possess two primary motivations: getting along (i.e., seeking acceptance in interpersonal relationships) and getting ahead (i.e., achieving status among a group of people). Self-monitoring is associated with the inclination to both (Day & Schleicher, 2006), which are critical factors influencing leadership emergence (Judge et al., 2009; Marinova et al., 2013). Finally, compared to LSMs, HSMs are more inclined to exhibit leader behaviors (Eby et al., 2003), including *initiating structure*, that is, clarifying roles and specifying rules and procedures (e.g., Dobbins et al., 1990) and *consideration*, that is, demonstrating concern, respect, and support for followers (e.g., Hall et al., 1998). These leadership behaviors would contribute to their perception as leaders within the group (Badura et al., 2022).

***H1**. Self-monitoring is positively related to leadership emergence.*

Self-monitoring motivation encompasses both “getting ahead” and “getting along,” which are two important drives leading to leadership emergence (Badura et al., 2022; Judge et al., 2009; Marinova et al., 2013). In addition, the acquisitive motivation of self-monitoring involves the status-enhancement motive (Day & Schleicher, 2006), and being a leader entails having status over others. Thus, the acquisitive motive aspect becomes critical for actively engaging in self-monitoring, including positive image-building and engagement in leader behaviors. Consequently, when self-monitoring is measured by scales that include self-monitoring motivation (i.e., SMS and SMS-R), its relationship with leadership emergence is expected to be stronger compared to when it is measured by RSMS, which excludes the motivation component.

***H2**. Self-monitoring scale moderates the relationship between self-monitoring and leadership emergence, such that the relationship is stronger when self-monitoring is measured by (**H2a**) SMS and (**H2b**) SMS-R as compared to when it is measured by RSMS.*

Leadership emergence has been measured in two ways. First, by subjective perceptions of others (e.g., peers, observers, and subordinates^1^), which are measured by ratings, such as using the *General Leadership Impression* (GLI) scale (e.g., Kilduff et al., 2017). Second, by peers either nominating (Kalish & Luria, 2021) or ranking (Zaccaro et al., 1991) whom they perceive to be emergent leaders. We expect a stronger relationship between self-monitoring and leadership emergence when the latter is measured by peers rather than subordinates. When interacting with subordinates, leaders may feel a reduced need to actively cultivate their image to enhance their status, likely because their status is already solidified through formal leadership positions (Badura et al., 2022). Conversely, when interacting with peers where individuals have not recognized formal status or authority, they are more likely to engage in self-monitoring to elevate their standing. Furthermore, compared to peers, subordinates are often seen as lower on the hierarchical ladder and might be deemed less relevant to instrumental goals. As a result, self-monitoring will be more activated to cultivate a more positive image when interacting with peers than subordinates, resulting in a stronger relationship between self-monitoring and leadership emergence when the latter is measured by peers than subordinates.

***H3**. Rating source moderates the relationship between self-monitoring and leadership emergence, such that the relationship is stronger when leadership is measured by peers’ ratings as compared to when it is measured by subordinates’ ratings.*

### Leadership Effectiveness

*Leadership effectiveness* (or *leader effectiveness*, e.g., Hoffman et al., 2011) refers to “a leader’s performance in influencing and guiding the activities of his or her unit toward the achievement of its goals” (Judge et al., 2002, p. 767). Even though leadership effectiveness is an individual-level construct, Hogan et al. (1994) suggested that it should be measured based on the unit: the group, team, or organization’s effectiveness as led by the leader. However, in practice, leadership effectiveness is usually measured through subjective ratings, including assessments from the leader’s supervisor, peers, or subordinates (Judge et al., 2002). These subjective ratings of leadership effectiveness are influenced by followers’ implicit leadership theories, which suggest that individuals tend to perceive those who are dedicated, charismatic, intelligent, and sensitive as effective leaders (Offermann et al., 1994).

Self-monitoring could positively contribute to leadership effectiveness for three reasons. First, compared to LSMs, HSMs are more sensitive and responsive to others’ reactions and expectations. This enables HSMs to better discern and consider followers’ needs and feedback (Bedeian & Day, 2004), contributing to the leading process (George, 2000). Moreover, followers who perceive their leaders as sensitive are more likely to consider them effective based on implicit leadership theory (Offermann et al., 1994). Second, HSMs’ greater behavioral flexibility in different situations helps them lead the group to respond to various situational demands and adapt to changes (Foti & Hauenstein, 2007; Zaccaro et al., 1991). This flexibility is an important aspect of effective leadership (Zaccaro et al., 1991). Finally, self-monitoring is positively associated with extraversion, openness, and emotional intelligence (Barrick et al., 2005; Wilmot et al., 2016), which are important attributes for leadership effectiveness (Judge et al., 2004; Rosete & Ciarrochi, 2005).

However, self-monitoring could also have negative effects on leadership effectiveness, including the following aspects. First, HSMs’ chameleon-like character and behavioral inconsistency may make them less trustworthy to followers compared to principled and consistent LSMs (Tasselli & Kilduff, 2018). Reduced trust in the leader could hinder leader-follower interactions and lead to negative attitudes, such as lower follower commitment and job satisfaction (Dirks & Ferrin, 2002), ultimately undermining leadership effectiveness. Second, HSMs may tend to be opportunistic in advancing their status (Oh et al., 2014), leading to unethical decision-making (Ogunfowora et al., 2013) and counterproductive behaviors toward organizations (Parks & Mount, 2005). These actions could diminish their credibility as leaders, negatively impacting the leading process and overall effectiveness of the unit. Finally, HSMs show a lower level of both attitudinal and behavioral commitment to interpersonal relationships and organizations compared to LSMs (Day et al., 2002). Therefore, leaders who are HSMs tend to be less committed than LSMs, which could have a negative impact on team or organizational outcomes. A lower level of commitment and dedication may also influence the subjective perception of leadership effectiveness (Offermann et al., 1994).

Self-monitoring appears to have both positive and negative aspects on leadership effectiveness. However, some of the negatives are more relevant to the unit’s objective performance. Since leadership effectiveness is usually measured through subjective ratings by others, and self-monitoring overall concerns creating a positive impression on others, we would hypothesize that the overall relationship (main effect) between self-monitoring and leadership effectiveness is positive.

***H4.** Self-monitoring is positively related to leadership effectiveness.*

The aforementioned positive associations between self-monitoring and leadership effectiveness are largely relevant to self-monitoring abilities, namely, “sensitivity to the expressive behaviors of others” and “ability to modify self-presentation” (Lennox & Wolfe, 1984, p. 1361). These abilities enable leaders to have the sensitivity to attend to followers’ needs and the behavioral flexibility to adapt to situational demands and changes. However, it is the self-serving instrumental motive of self-monitoring that would lead to inconsistent, opportunistic, and even unethical behaviors, which could result in a leader’s reduced credibility and harm to the team or organization. Thus, when self-monitoring is measured by the RSMS (a scale that focuses on self-monitoring ability and excludes self-monitoring motivation), the relationship with leadership effectiveness would be stronger than when it is measured by SMS and SMS-*R* (scales that include self-monitoring motivation).

***H5.** Self-monitoring scale moderates the positive relationship between self-monitoring and leadership effectiveness, such that the relationship is stronger when self-monitoring is measured by RSMS as compared to when it is measured by (**H5a**) SMS and (**H5b**) SMS-R.*

We expect that self-monitoring will show a stronger positive correlation with leadership effectiveness when the latter is rated by supervisors rather than by subordinates, for two reasons. First, compared to leaders who are LSMs, HSMs are more likely to demonstrate more positive performance in front of supervisors than subordinates since the former is more critical to HSMs’ instrumental purposes. This aligns with the finding that HSMs tend to behave more positively in interpersonal settings where their behaviors are visible to supervisors, compared to situations where their behaviors are not witnessed by important audiences (e.g., Oh et al., 2014). Second, leaders are expected to interact more frequently with their subordinates than with their supervisors in the leading process. As a result, subordinates are more likely to observe HSMs’ negative aspects, such as inauthenticity, inconsistent behaviors, and unethical conduct, which could lead them to rate the leader less favorably. In contrast, supervisors may have fewer opportunities to witness such negative behaviors and may only see the more positively cultivated side of HSMs during interactions, resulting in more favorable ratings of leadership effectiveness.

***H6**. Rating source moderates the relationship between self-monitoring and leadership effectiveness, such that the relationship is stronger when leadership is measured by supervisors’ ratings as compared to when it is measured by subordinates’ ratings.*

### Authentic Leadership

Authentic leadership is grounded in the construct of *authenticity*, which traces back to the ancient Greek philosophy of “to thine own self be true” (Gardner et al., 2011). Authenticity refers to “consistency between an entity’s external expressions and its internal values and beliefs” (Lehman et al., 2019, p. 1). Built on the conceptualization of authenticity, authentic leadership is defined as

“a pattern of leader behavior that draws upon and promotes both positive psychological capacities and a positive ethical climate, to foster greater self-awareness, an internalized moral perspective, balanced processing of information, and relational transparency on the part of leaders working with followers, fostering positive self-development” (Walumbwa et al., 2008, p. 94).

Authentic leadership incorporates four dimensions: (a) self-awareness, (b) balanced processing, (c) relational transparency, and (d) internalized moral perspective (Gardner, Avolio, et al., 2005; Walumbwa et al., 2008).

Self-monitoring contradicts two of these components of authentic leadership, that is, relational transparency (the third component) and internalized moral perspective (the fourth component). *Relational transparency* requires leaders to establish a transparent relationship with followers by demonstrating their true selves in leader-follower interactions (Avolio & Gardner, 2005; Shamir & Eilam, 2005). However, leaders with a higher level of self-monitoring may hide their true feelings and inner states, and this would undermine the development of a transparent relationship between leaders and followers. *Internalized moral perspective* requires leaders to have high moral and ethical standards, according to which they act in a principled manner (Luthans & Avolio, 2003; May et al., 2003). However, self-monitoring is associated with unethical behaviors such as unethical business decision-making (Ogunfowora et al., 2013), faking in interviews (Levashina & Campion, 2007), and counterproductive behaviors toward organizations (Parks & Mount, 2005), which conflicts with this component. Also, under situational pressure, HSMs tend to behave in a pragmatic manner, in contrast to authentic leaders who would behave in a manner consistent with their internalized values (Walumbwa et al., 2008). Thus, we expect self-monitoring to be negatively related to authentic leadership.

Also, self-monitoring involves adjusting self-presentation to meet situational demands rather than acting according to the inner self (Gangestad & Snyder, 2000) and therefore contradicts the very essence of authenticity, that is, consistency between one’s expressive behaviors and inner true self (Lehman et al., 2019). Indeed, empirical studies find a negative correlation between self-monitoring and authenticity (Brotheridge & Lee, 2002; Pillow et al., 2017). Research also finds that HSMs, as compared to LSMs, demonstrate a lower level of intention-behavior consistency (Ajzen et al., 1982) and are more engaged in surface acting (Brotheridge & Lee, 2002), both of which imply low authenticity. According to Gardner, Avolio, et al. (2005, p. 345), “First and foremost, an authentic leader must achieve authenticity.” Hence, we expect self-monitoring to be negatively related to authentic leadership.

***H7.** Self-monitoring is negatively related to authentic leadership.*

The factor influencing inauthenticity is the self-monitoring motivation, not self-monitoring abilities including “sensitivity to the expressive behaviors of others” and “ability to modify self-presentation” (Lennox & Wolfe, 1984, p. 1361). When individuals are driven by the desire to strategically manage impressions for personal gain, they could risk compromising their authenticity. Thus, the negative association between self-monitoring and authentic leadership stems from the motivation to self-monitor, rather than the ability to do so, and the two self-monitoring abilities alone do not result in one’s inauthenticity.

These two abilities could, conversely, contribute to authentic leadership as they could be associated with two components of authentic leadership: *self-awareness* and *balanced processing*. Self-awareness involves recognizing and comprehending one’s emotions (Ilies et al., 2005), an integral aspect of emotional intelligence (EI), which encompasses the ability to perceive, understand, manage, and effectively utilize emotions (Salovey & Mayer, 1990). Research shows a positive correlation between self-monitoring ability (measured by RSMS) and EI (e.g., Wilmot, 2011), suggesting that self-monitoring ability is likely to enhance the authentic leadership dimension of self-awareness. *Balanced processing* refers to objectively perceiving self-relevant information, including reactions and feedback from others (Ilies et al., 2005). It requires leaders to perceive information from different perspectives and to sense cues and information from various people and situations (Avolio & Gardner, 2005). This requires the self-monitoring ability of sensitivity. Therefore, since RSMS is solely focused on self-monitoring ability, but SMS and SMS-R involve the motivation to self-monitor, the dimension contributing to inauthenticity, we hypothesize the measurement scale of self-monitoring would moderate the relationship between self-monitoring and authentic leadership.

***H8.** Self-monitoring scale moderates the relationship between self-monitoring and authentic leadership, such that the relationship is positive when self-monitoring is measured by RSMS as compared to a negative relationship when it is measured by SMS-R.^2^*

### Transformational Leadership

Transformational leadership refers to “the leader moving the follower beyond immediate self-interests through idealized influence (charisma), inspiration, intellectual stimulation, or individual consideration” (Bass, 1999, p. 11). Transformational leadership thus incorporates four dimensions, that is, (a) idealized influence, (b) inspirational motivation, (c) intellectual stimulation, and (d) individualized consideration (Avolio & Bass, 2002; Bass, 1985). Self-monitoring can positively impact transformational leadership for the following three reasons. First, self-monitoring involves constructing a positive self-image in various situations, which seems to be linked to the charisma of the leader (Gardner & Avolio, 1998). Second, self-monitoring concerns sensing and understanding other people’s needs, feelings, and reactions, which helps the leader influence and develop followers—a key aspect of transformational leadership in transforming followers to go beyond self-interest toward a shared vision (Gardner & Avolio, 1998; Siangchokyoo et al., 2020). Finally, self-monitoring involves adaptability to different environments, which aids leaders in guiding the team or organization toward positive changes—a broad goal of transformational leadership (Eisenbach et al., 1999; Sosik et al., 2002).

However, self-monitoring can be negatively associated with the first two dimensions of transformational leadership. *Idealized influence* refers to the leader influencing followers by being a role model (Bass, 1999). The leader is influential in promoting ideals (Bass, 1999), where he or she transcends self-interests to lead a group or organization toward greatness and influences followers to transcend their self-interests for this common goal (Bass, 1985; Siangchokyoo et al., 2020). The leader also places followers’ needs over self-needs in the influential process (Bass et al., 2003). Inherent in this dimension is the leader’s commitment to personal values, principles, and morality (Bass, 1999; Walumbwa et al., 2008), based on which the leader serves as a role model, inspiring followers to transform their own values and beliefs accordingly (Kuhnert & Lewis, 1987). However, self-monitoring is motivated by self-serving goals to enhance status in front of others (Fuglestad & Snyder, 2010) rather than place others’ needs and common goals beyond self-interest, and self-monitoring involves pragmatic actions toward situations rather than acting based on principles. Thus, self-monitoring contradicts this dimension. *Inspirational motivation* refers to motivating followers by providing meaning to work and articulating a clear vision to followers (Bass, 1985). This dimension is highly correlated with idealized influence (Judge & Bono, 2000), as leaders inspire followers to trust in the envisioned future by setting themselves as an example. These leaders firmly believe in the vision and behave consistently with their beliefs (Bass, 1999; Bass et al., 2003). Therefore, inspiring followers is built on leaders’ deeply held principles and authentic behaviors, which contradicts chameleon-like HSMs.

Another aspect that the relationship between self-monitoring and transformational leadership can be negative is the conceptual overlap between transformational leadership and authentic leadership (Luthans & Avolio, 2003; Walumbwa et al., 2008). This overlap has been confirmed by empirical studies (Banks et al., 2016; Spitzmuller & Ilies, 2010). For instance, Banks et al.’s (2016) meta-analysis shows a strong correlation between transformational leadership and authentic leadership.

Self-monitoring could both contribute to and undermine transformational leadership. However, the essential dimension of transformational leadership, idealized influence, is grounded in principled and values-driven behaviors (Bass, 1999). The chameleon-like nature of self-monitoring conflicts with this fundamental dimension. In addition, transformational leadership overlaps with authentic leadership (Walumbwa et al., 2008), which emphasizes authenticity, contradicting the nature of self-monitoring. Given these, we hypothesize an overall negative relationship between self-monitoring and transformational leadership.

***H9**. Self-monitoring is negatively related to transformational leadership.*

### Transactional Leadership

Transactional leadership refers to “the exchange relationship between leader and follower to meet their own self-interests” (Bass, 1999, p. 10). Compared to transformational leaders who inspire followers to pursue a vision beyond self-interests, transactional leadership involves catering to followers’ immediate self-interests, expectations, and needs (Bass, 1999; Kuhnert & Lewis, 1987). Transactional leadership comprises three dimensions: (a) contingent rewards—providing rewards for achieving performance goals, (b) management by exception—intervening when deviations occur, and (c) laissez-faire—adopting a hands-off approach, allowing followers to manage tasks independently (Bass et al., 2003).

The core of transactional leadership is *contingent rewards*, in which the leader specifies and clarifies tasks and requirements for followers, including the rewards followers could attain by achieving requirements (Bass, 1985). Self-monitoring is expected to be positively related to transactional leadership for the following two reasons. First, the core of transactional leadership, contingent reward, relies on leaders’ accurate understanding of followers’ interests, expectations, and needs, by which leaders could persuade followers to accomplish tasks in exchange for fulfilling followers’ demands. Followers’ demands include tangible rewards such as money and resources, and intangible rewards such as recognition and praise (Bass et al., 2003). Self-monitoring involves accurately sensing and understanding others’ expectations, which would help the leader understand what to provide to followers so that they would best accomplish work. Second, self-monitoring involves a pragmatic manner in the interaction with others, which aligns with transactional leaders’ tendency to lead followers based on the exchange of interests. Therefore, we hypothesize the following:

***H10**. Self-monitoring is positively related to transactional leadership.*

### Managerial Leadership

Managerial leadership involves supervising subordinates to accomplish tasks and managing daily operations (Bedeian & Hunt, 2006; Yukl, 1989). It focuses on overseeing followers’ day-to-day responsibilities at the micro-level and does not extend to developing followers or leading macro-level organizational changes (Bedeian & Hunt, 2006; Hunt, 2004). The tasks managed by managerial leaders are often diverse, fast-paced, and fragmented (Yukl, 1989). To effectively handle these complex tasks and roles, leaders need to demonstrate a combination of managerial and leadership behaviors, encompassing both task-oriented and relationship-oriented approaches, rather than focusing on a single type of leader behavior (Denison et al., 1995; Lawrence et al., 2009; Yukl, 1989). The concept of managerial leadership aligns with the theory of managerial grid (Blake et al., 1962), which suggests that effective managers should display concern for both production and people, resembling task-oriented and relationship-oriented leadership behaviors (Bernardin & Alvares, 1976).

Regarding leadership behaviors, the early behavioral approach by Bales (1950) categorized leader behaviors into *task* and *socio-emotional* functions (Lord et al., 2017). Meanwhile, a group of researchers at Ohio State University examined day-to-day leadership behaviors (Fleishman, 1953; Stogdill, 1950) and found that the behaviors leaders engage in could be grouped into two broad categories: *initiating structure* (or structure) and *consideration* (Fleishman, 1953). These two categories align with Bales’ task and socio-emotional behaviors, respectively (Lord et al., 2017), and scholars later categorized and studied these behaviors as *task-oriented* and *relationship-oriented* behaviors, respectively (Derue et al., 2011).

We hypothesize a positive relationship between self-monitoring and managerial leadership. This is supported by research indicating that self-monitoring is positively associated with general leader behaviors (Church, 1997; Day et al., 2002) as well as specific behaviors, including both task-oriented (e.g., Dobbins et al., 1990; Eby et al., 2003) and relationship-oriented behaviors (e.g., Hall et al., 1998). Given the day-to-day complexity and reactivity of managerial work, effective managerial leaders need to exhibit both task-oriented and relationship-oriented behavioral styles to adapt to the diverse requirements of followers and situations (Yukl, 1989). Therefore, we hypothesize the following:

***H11.** Self-monitoring is positively related to managerial leadership.*

Regarding the moderating role of rating sources, we hypothesize that supervisors would rate managerial leaders more positively than subordinates. First, compared to LSMs, HSMs are expected to exhibit superior managerial behaviors in the presence of supervisors, driven by their instrumental motives. Second, especially with regard to relationship-oriented behaviors, HSMs are anticipated to manifest these behaviors more toward their supervisors. This encompasses showing enhanced respect, extending more help, and furnishing augmented support, with the aim of securing advantages from their supervisors. Therefore, we hypothesize the following:

***H12**. Rating source moderates the relationship between self-monitoring and managerial leadership, such that the relationship is stronger when leadership is measured by supervisors’ ratings as compared to when it is measured by subordinates’ ratings.*

## Method^3^

### Collection of Sample

**Figure 1.**
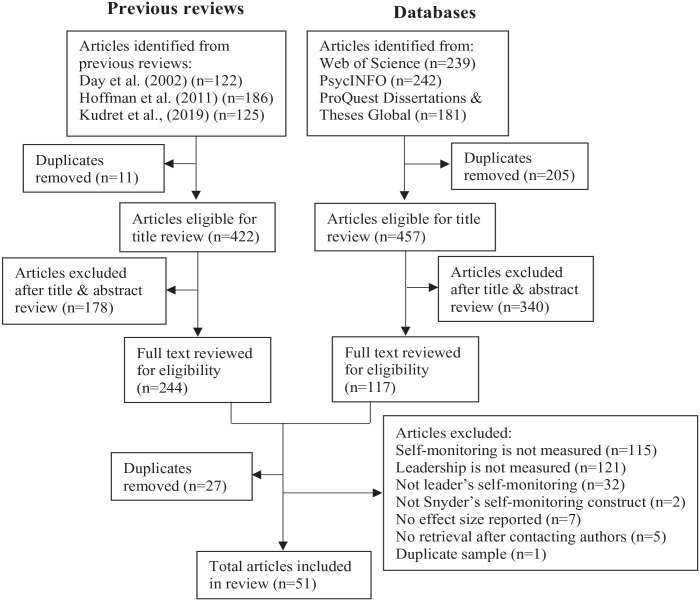
PRISMA Flow Diagram for Final Article Inclusion. *Note.* Articles excluded after title & abstract review include the following apparent reasons: not the self-monitoring concept developed by Snyder (1974), such as “self-monitoring” in computer, biology, health care, etc.; non-empirical paper; not at all relevant to leadership. Kudret et al. (2019) examined post-2000 studies, Day et al. (2002) focused on leadership emergence and behavior, and Hoffman et al. (2011) concentrated on leadership effectiveness. The three reviews included studies on self-monitoring and leadership, along with a list of other variables. This is why they only have a few duplicates, and many studies could be excluded after title and abstract screening.

Our search methods were in line with the Preferred Reporting Items for Systematic Reviews and Meta-Analyses (PRISMA) guidelines (Page et al., 2021). The search and screening procedure is displayed in Figure 1. First, we conducted searches in Web of Science, PsycINFO, and ProQuest Dissertations & Theses Global, covering the period from 1974 (when the self-monitoring construct was introduced) until July 2023. We used combined keywords of self-monitor* AND (leader* OR manager* OR supervisor OR follower* OR subordinate) appearing in the abstract. Second, we searched for studies included in previous review papers that examined the relationship between self-monitoring and leadership (Day et al., 2002; Hoffman et al., 2011; Kudret et al., 2019). Based on the PRISMA guidelines, the initial results were screened by the first two authors to assess their eligibility. After completing a full-text review, 51 articles (55% journal articles, 41% dissertations, and 4% book chapters), which include 55 independent samples (some articles contain multiple samples, whereas several articles used identical samples), meet the inclusion criteria. The total sample size across articles is 9029.

Articles were considered eligible for inclusion based on the following criteria. First, the study’s “self-monitoring” variable aligns with Snyder’s (1974) definition, which refers to “self-observation and self-control guided by situational cues to social appropriateness” (p. 526). Studies focusing on other forms of “self-monitoring” unrelated to this definition, such as family “self-monitoring” of business (Ghaleb et al., 2020), “self-monitoring” of diet and exercise (Campbell & Crawford, 2000), were excluded. Second, since our research question concerns the impact of a leader’s self-monitoring on leadership outcomes, the study must assess the leader’s self-monitoring, excluding studies that only measured followers’ self-monitoring. Third, the study should report at least one effect size for the zero-order correlation (Day et al., 2002) between self-monitoring and any leadership variables. Alternatively, the study should have provided data that allow for the computation of a zero-order correlation, such as Cohen’s *d* or *t*-statistic. Finally, to conduct a meta-analysis of the relationship, there should be a minimum of two studies (i.e., *k* ≥ 2) that present the relationship between self-monitoring and a leadership-dependent variable.

### Coding

The first two authors examined the studies in the sample and together decided on the coding protocols. After deciding the protocol, the first author coded all the articles, including sample size, correlations, and reliabilities of self-monitoring and leadership variables. The first author cross-checked the coding at least three times: during the coding process, when calculating composite scores, and during the final stage of analyzing data. The second author and a research assistant then checked all the coding for each article and recorded the agreement of coding. The final interrater consistency was 97%. Whenever there were questions and uncertainties, the first and second authors discussed to reach an agreement.

### Leadership Variables

For *leadership emergence*, we included studies that explicitly operationalized leadership emergence and those that regarded leadership perception as equivalent to leadership emergence (e.g., Hall et al., 1998). Regarding *leadership effectiveness*, we included studies that specifically operationalized leadership effectiveness; following Hoffman et al. (2011), we grouped leader performance and managerial performance under this category. We also included leader’s job promotion, given that promotion decisions are contingent on leadership effectiveness (Giessner & van Knippenberg, 2008).

For *authentic leadership*, we included studies that used the following measures: the Authentic Leadership Questionnaire (ALQ) (Walumbwa et al., 2008), the Authentic Leadership Inventory (ALI) (Neider & Schriesheim, 2011), or an originally developed scale by Tate (2008). Studies that measured transformational and transactional leadership using the Multifactor Leadership Questionnaire (MLQ) (Bass & Avolio, 1996) were included under the respective leadership variables. Finally, drawing from Yukl (1989), we categorized managerial leadership as task-oriented leader behaviors (e.g., initiating structure), relationship-oriented leader behaviors (e.g., consideration), and generic leadership behaviors (i.e., behaviors not specifically categorized into either task- or relationship-oriented behavior by the original authors). This categorization aligns with Day et al.’s (2002) practice of grouping various leader behaviors into a broad leadership behavior category.

### Meta-Analytical Procedures

We applied the Schmidt-Hunter random-effect meta-analysis method (Hunter & Schmidt, 1990), which enabled us to produce a sample-weighted mean correlation (*r*) and a mean correlation corrected for unreliability (*ρ*). The majority of studies in the sample reported internal reliability of variables (i.e., Cronbach’s alpha). For studies that reported reliabilities at the facet level, we computed composite reliabilities for the overall construct using Mosier’s (1943) formula. For studies that did not report internal reliability, we imputed the average Cronbach’s alpha of other studies included in this meta-analysis (Hunter & Schmidt, 1990).

We used the “metafor” package (Viechtbauer, 2010) in R to conduct the analyses. We reported 95% confidence intervals (CI) and 80% credibility intervals (CV). CI provides the level of uncertainty around the estimated mean correlation due to sampling error (Whitener, 1990). 95% CI indicates that we can be 95% certain that the corrected mean correlation (*ρ*) lies in the lower and upper limits of the interval. The corrected correlation is considered to be statistically significant if the 95% confidence interval does not include zero. CV provides the range of values by which the estimated mean correlations (*ρ*) can vary due to moderators (Whitener, 1990). 80% CV indicates that there is an 80% probability that the corrected mean correlation (*ρ*) would lie within this interval.

We first implemented Egger’s regression test (Egger et al., 1997) to assess publication bias. A significant intercept indicates asymmetry in the funnel plot and the possibility for publication bias (Banks et al., 2012; Webster, 2019). When statistically significant results were observed in Egger’s regression test, we used a trim-and-fill procedure to obtain a bias-corrected estimate of the overall effect (Duval, 2005; Duval & Tweedie, 2000a, 2000b). Funnel plots for the main effects are presented in online supplemental materials.

## Results

### Main Effects

Table 1 presents the main effects of self-monitoring and leadership variables. The relationship between self-monitoring and leadership emergence is positive (*ρ* = .171, 95% CI [.110, .232]), supporting H1. Self-monitoring is also positively related to leadership effectiveness (*ρ* = .123, 95% CI [.061, .184]), supporting H4.

**Table 1.** Self-Monitoring and Leadership: Main Effects.

Leadership variables	*k*	*N*	*r*	*SD_r_*	*ρ*	*SD_ρ_*	95% CI		80% CV	
							*LL*	*UL*	*LL*	*UL*
Leadership Emergence	25	3,355	.145	.120	**.171**	.031	.110	.232	.046	.296
Leadership Effectiveness	25	3,162	.099	.116	**.123**	.032	.061	.184	.010	.235
Authentic Leadership	5	594	.199	.149	**.248**	.100	.053	.444	.047	.449
Transformational Leadership	7	829	.079	.085	**.094**	.043	.010	.179	.094	.094
Transactional Leadership	3	395	.002	.096	−.005	.074	−.150	.141	−.069	.059
Managerial Leadership	18	3,802	.095	.086	**.121**	.035	.053	.190	.047	.196
Task-oriented Behavior	12	1,264	.169	.107	**.205**	.039	.129	.281	.133	.278
Relationship-oriented Behavior	13	1,308	.078	.116	**.100**	.042	.018	.182	.006	.193

*Note.* Effects in **bold** are statistically significant. Managerial Leadership includes task-oriented and relationship-oriented behaviors and generic leadership behaviors not categorized as either task/relationship-oriented behavior by the original author(s). *k* = number of samples; *N* = total sample size; *r* = sample-size weighted average correlation; *SD*_r_ = standard deviation of sample-size weighted average correlation; *ρ* = estimated true-score correlation; *SD_ρ_* = standard deviation of estimated true-score correlation; 95% CI = 95% confidence interval; 80% CV = 80% credibility interval; *LL* = lower limit; *UL* = upper limit.

Self-monitoring is positively related to authentic leadership (*ρ* = .248, 95% CI [.053, .444]), not supporting H7. Self-monitoring is positively related to transformational leadership (*ρ* = .094, 95% CI [.010, .179]), not supporting H9. The relationship between self-monitoring and transactional leadership is non-significant (*ρ* = −.005, 95% CI [−.150, .141]), not supporting H10. The relationship between self-monitoring and managerial leadership is positive (*ρ* = .121, 95% CI [.053, .190]), supporting H11. With regard to specific leadership behaviors, self-monitoring is positively related to both task-oriented behavior (*ρ* = .205, 95% CI [.129, .281]) and relationship-oriented behavior (*ρ* = .100, 95% CI [.018, .182]).

### Moderator Analyses

Table 2 presents the moderation effects of different self-monitoring scales. The correlation between self-monitoring and leadership emergence is statistically significant when self-monitoring is measured using RSMS (*ρ* = .175, 95% CI [.099, .250]) and SMS-*R* (*ρ* = .266, 95% CI [.165, .367]) but not SMS (*ρ* = .086, 95% CI [−.006, .178]). Further *z* tests (Raju & Brand, 2003) suggest that the effect size is significantly weaker when self-monitoring is measured using SMS than SMS-*R* (*z* = −2.613, *p* < .05). There was no statistical significance when comparing RSMS with SMS (*z* = −1.683, *p* > .05) or RSMS with SMS-*R* (*z* = −1.657, *p* > .05). Therefore, H2a and H2b are not supported.

**Table 2.** Moderation Effects of Self-Monitoring Measurement Scale.

Leadership variables	*k*	*N*	*r*	SD_r_	*ρ*	*SD_ρ_*	95% CI		80% CV		*z* test
							LL	UL	LL	UL	
Leadership Emergence											
RSMS^a^	17	2,421	.151	.122	**.175**	.038	.099	.250	.044	.305	1.683^ab^
SMS^b^	5	705	.069	.078	.086	.047	−.006	.178	.086	.086	−2.613**^bc^
SMS-R^c^	5	517	.218	.063	**.266**	.052	.165	.367	.266	.266	−1.657^ac^
Leadership Effectiveness											
RSMS^a^	9	1,638	.147	.085	**.175**	.036	.104	.246	.109	.240	**3.712** *** ^ab^
SMS^b^	9	669	−.028	.137	−.036	.059	−.152	.080	−.148	.078	−2.602**^bc^
SMS-R^c^	7	855	.107	.065	**.131**	.042	.049	.213	.131	.131	.872^ac^
Authentic Leadership											
RSMS^a^	2	370	.302	.028	**.391**	.061	.270	.511	.391	.391	
SMS-R^c^	2	166	.032	.128	.048	.109	−.165	.261	−.046	.142	
Transformational Leadership											
SMS^b^	3	210	.058	.068	.073	.092	−.108	.254	.073	.073	−1.129^bc^
SMS-R^c^	3	471	.129	.054	**.159**	.057	.048	.270	.159	.159	
Managerial Leadership											
RSMS^a^	8	1,061	.182	.094	**.215**	.039	.138	.292	.159	.272	**3.356** *** ^ab^
SMS^b^	6	2,207	.052	.024	**.067**	.028	.013	.121	.067	.067	−1.179^bc^
SMS-R^c^	6	822	.102	.084	**.127**	.043	.042	.212	.127	.127	1.606^ac^

*Note.* Effects in **bold** are statistically significant. We only conducted analysis when at least two studies reported the correlation, and some subcategory analysis cannot be implemented. The addition of subcategory *k* can be larger than the total *k* because some studies include more than one subcategory datapoint. *k* = number of samples; *N* = total sample size; *r* = sample-size weighted average correlation; *SD*_r_ = standard deviation of sample-size weighted average correlation; *ρ* = estimated true-score correlation; *SD_ρ_* = standard deviation of estimated true-score correlation; 95% CI = 95% confidence interval; 80% CV = 80% credibility interval; LL = lower limit; UL = upper limit; *z* test = tests whether effect sizes are significantly different; RSMS = Revised Self-monitoring Scale; SMS= Self-monitoring Scale; SMS-R = Shorter version of SMS.

ab *z* test between self-monitoring measured by RSMS and SMS. ^bc^ *z* test between self-monitoring measured by SMS and SMS-R. ^ac^ *z* test between self-monitoring measured by RSMS and SMS-R.

* *p* < .05. **p < .01. ***p < .001.

H5 proposes that the relationship between self-monitoring and leadership effectiveness will be stronger when self-monitoring is measured by RSMS than (H5a) SMS and (H5b) SMS-R. The correlations are positive and significant when self-monitoring is measured using RSMS (*ρ* = .175, 95% CI [.104, .246]) and SMS-*R* (*ρ* = .131, 95% CI [.049, .213]) but non-significant using SMS (*ρ* = −.036, 95% CI [−.152, .080]). Further examination suggests that the effect is significantly stronger when self-monitoring is measured with RSMS than SMS (*z =* 3.712, *p* < .05), supporting H5a. However, no such significant differences were observed when self-monitoring is measured by RSMS versus SMS-*R* (*z* = .872, *p* > .05), not supporting H5b. In addition, there is a statistically significant difference between when self-monitoring is measured by SMS and SMS-*R* (*z =* −2.602, *p* < .05).

H8 hypothesized that the relationship between self-monitoring and authentic leadership is significantly different when self-monitoring is measured by RSMS as compared to SMS-R. The correlation is positive and significant when self-monitoring is measured by RSMS (*ρ* = .391, 95% CI [.270, .511]), whereas the correlation is non-significant when self-monitoring is measured by SMS-*R* (*ρ* = .048, 95% CI [−.165, .261]). We were not able to conduct a *z* test because of the small number of independent studies. However, the CI between these two effect sizes did not overlap, providing preliminary evidence that the type of self-monitoring scale may be a moderator.

Table 3 presents the moderation effects of different rating sources. The correlation between self-monitoring and leadership emergence is positive for peer-rating (*ρ* = .158, 95% CI [.084, .232]), observer-rating (*ρ* = .178, 95% CI [.045, .312]) and self-rating (*ρ* = .269, 95% CI [.082, .456]), but non-significant for subordinate-rating (*ρ* = .135, 95% CI [−.064, .334]). Results from a series of *z* tests show that there were no statistically significant differences among these effect sizes. Therefore, H3 is not supported.

**Table 3.** Moderation Effects of Leadership Rating Source.

Leadership variables	*k*	*N*	*r*	SD_r_	*ρ*	*SD_ρ_*	95% CI		80% CV		*z* test
							LL	UL	LL	UL	
Leadership Emergence											
Subordinate^a^	2	174	.112	.121	.135	.102	−.064	.334	.053	.217	
Peer^b^	19	2,568	.134	.125	**.158**	.038	.084	.232	.022	.294	−.250^ab^
Observer^c^	3	425	.148	.103	**.178**	.068	.045	.312	.110	.246	−.407^ac^, −.328^bc^
Self^d^	3	259	.213	.127	**.269**	.095	.082	.456	.144	.395	−1.267^ad^, −1.434^bd^, −.977^cd^
Leadership Effectiveness											
Subordinate^a^	9	1,075	.024	.120	.034	.049	−.063	.131	−.082	.150	
Self^d^	5	1,222	.184	.086	**.223**	.051	.124	.323	.131	.314	−3.780***^ad^
Supervisor^e^	13	1,323	.056	.128	.071	.046	−.019	.160	−.057	.198	−.733^ae^, 3.157**^de^
Authentic Leadership											
Subordinate^a^	3	455	.215	.114	**.261**	.098	.070	.452	.109	.413	
Self^d^	3	197	.109	.202	.138	.150	−.155	.432	−.126	.403	1.181^ad^
Transformational Leadership											
Subordinate	7	829	.054	.071	.064	.044	−.021	.150	.064	.064	
Transactional Leadership											
Subordinate	3	395	.002	.096	−.005	.074	−.150	.141	−.069	.059	
Managerial Leadership											
Subordinate^a^	8	675	.046	.151	.058	.075	−.089	.204	−.110	.225	
Peer^b^	4	463	.220	.107	**.272**	.059	.157	.386	.213	.332	−3.007**^ab^
Observer^c^	5	2,481	.072	.057	**.093**	.040	.015	.172	.040	.146	−.704^ac^, 3.048**^bc^
Self^d^	8	748	.030	.115	.041	.053	−.064	.145	−.035	.116	.296^ad^, 3.288**^bd^, 1.033^cd^
Supervisor^e^	3	402	.146	.020	**.184**	.062	.063	.305	.184	.184	−1.645^ae^, 1.108^be^, −1.372^ce^, −1.881^de^

*Note.* Effects in **bold** are statistically significant. *k* = number of samples; *N* = total sample size; *r* = sample-size weighted average correlation; *SD*_r_ = standard deviation of sample-size weighted average correlation; *ρ* = estimated true-score correlation; *SD_ρ_* = standard deviation of estimated true-score correlation; 95% CI = 95% confidence interval; 80% CV = 80% credibility interval; LL = lower limit; UL = upper limit; *z* test = tests whether effect sizes are significantly different. We only conducted analysis when at least two studies reported the correlation, and some subcategory analysis cannot be implemented. The addition of subcategory *k* can be larger than the total *k* because some studies include more than one subcategory datapoint. The rating source indicates rating by this source only, excluding those with multiple rating sources. We only conducted analysis when at least two studies reported the correlation, and some subcategory analysis cannot be implemented. The addition of subcategory *k* can be larger than the total *k* because some studies include more than one subcategory datapoint. The rating source indicates rating by this source only, excluding those with multiple rating sources.

ab z test between subordinate and peer ratings. ^ac^ z test between subordinate and observer ratings. ^bc^ *z* test between peer and observer ratings. ^ad^ *z* test between subordinate and self-ratings. ^bd^ *z* test between peer and self-ratings. ^cd^ *z* test between observation and self-ratings. ^ae^ *z* test between subordinate and supervisor ratings. ^de^ *z* test between self and supervisor ratings. ^be^ *z* test between peer and supervisor ratings. ^ce^ *z* test between observer and supervisor ratings.

* *p* < .05. **p < .01. ***p < .001.

The correlation between self-monitoring and leadership effectiveness is non-significant when the latter is measured by either subordinates (*ρ* = .034, 95% CI [−.063, .131]) or supervisors (*ρ* = .071, 95% CI [−.019, .160]), and there were no statistically significant differences among these effect sizes (*z =* −.733, *p* > .05). Therefore, H6 is not supported. However, our exploratory analyses suggest that the self-monitoring—leadership effectiveness relationship is positive and significant when leadership effectiveness is self-rated (*ρ* = .223, 95% CI [.124, .323]). The moderation effect by rating sources of leadership effectiveness is significant when comparing subordinate—with self-ratings (*z =* −3.780, *p* < .05) and self with supervisor ratings (*z* = 3.157, *p <* .05).

Finally, the meta-correlation between self-monitoring and managerial leadership is weaker when managerial leadership is reported by subordinates (*ρ* = .058, 95% CI [−.089, .204]) than by supervisors (*ρ* = .184, 95% CI [.063, .305]); however, the difference was not statistically significant (*z* = −1.645, *p* > .05). Therefore, despite being in the predicted direction, the results do not support H12. In our exploratory analyses involving managerial leadership reported by other sources, we find that the self-monitoring—managerial leadership relationship is positive and significant when managerial leadership is reported by peers (*ρ* = .272, 95% CI [.157, .386]) and other observers (*ρ* = .093, 95% CI [.015, .172]). Comparisons of *z* test results indicate that the relationship between self-monitoring and managerial leadership is significantly weaker when managerial leadership is rated by subordinates than by peers (*z =* −3.007, *p* < .05). Furthermore, self-monitoring has a significantly stronger correlation with managerial leadership when its peer-rated than observer-rated (*z =* 3.048, *p* < .05) or self-rated (*z* = 3.288, *p* < .05).

### Publication Bias

The Egger’s test showed no significant funnel-plot asymmetry in all but two relationships: (1) the main effect between self-monitoring and leadership effectiveness original *ρ* = .123 (*k* = 25, 95% CI [.061, .184]), Egger’s test (*z* = −2.198, *p* < .05), T&F *ρ* = .182 (imputed *k* = 8, 95% CI [.108, .257]) and (2) the relationship between self-monitoring and subordinate-rated authentic leadership original *ρ* = .261 (*k* = 3, 95% CI [.070, .452]), Egger’s test (*z* = −2.745, *p* < .05), T&F *ρ* = .373 (imputed *k* = 2, 95% CI [.145, .600]). As shown in the trim and fill results, despite the slight change in effect sizes (i.e., increasing by .059 and .112 for the above-mentioned relationships, respectively), both relationships remain statistically significant, and interpretations of these effects also remain the same. Therefore, the potential impact of publication was likely minimal in our meta-analysis. Details on Egger’s test for additional relationships and funnel plots for main effects are presented in Online Supplements A and B.

## Discussion

This meta-analysis examined the relationship between self-monitoring and six leadership variables, that is, leadership emergence, leadership effectiveness, authentic leadership, transformational leadership, transactional leadership, and managerial leadership. We also investigated the influence of two moderators, that is, self-monitoring measurement scale and rating sources of leadership. The findings show that except for transactional leadership, self-monitoring appears to have a modest positive relationship (*ρ* ranges from .094 to .248) with each of the other five leadership variables. However, the relationship between self-monitoring and the leadership variables is generally non-significant when the latter is rated by subordinates. The relationships also differ as to what self-monitoring scale measured self-monitoring. These findings make significant contributions to the literature by providing a more nuanced understanding of the relationship between self-monitoring and leadership while also offering clearer insights into both constructs.

### The Relationship between Self-monitoring and Leadership

Our findings advance the understanding of the complex nature of the self-monitoring and leadership relationship, revealing that it is not as simple as research has shown. Aligning with earlier studies (e.g., Day et al., 2002; Hoffman et al., 2011), our results indicate that the main effect between self-monitoring and leadership variables is generally positive. However, the present findings suggest that self-monitoring is not associated with leadership *in the eyes of subordinates*, who are the followers, the key constituency of the leader (Siangchokyoo et al., 2020). The relationship between self-monitoring and five leadership-related variables (i.e., leadership emergence, leadership effectiveness, transformational leadership, transactional leadership, and managerial leadership) is non-significant when they are measured by subordinate ratings. This finding is particularly important because subordinate ratings of leadership are often considered more valid than other ratings (Hogan et al., 1994; Kim & Yukl, 1995). Therefore, the generally positive self-monitoring–leadership relationship should be interpreted cautiously, given that relationships assessed via subordinate ratings are non-significant.

In addition, our findings offer clarity over the controversial findings between self-monitoring and leadership effectiveness (Kudret et al., 2019) by demonstrating that self-monitoring is only positively related to self-rated leadership effectiveness, whereas ratings from subordinates and supervisors show no significant correlations and differ significantly from self-ratings. It is possible that the positive association with self-rated leadership effectiveness is due to the potential narcissistic aspect of self-monitoring and its probable association with narcissistic leadership (Judge et al., 2006; Rosenthal & Pittinsky, 2006). Indeed, prior research indicates that narcissists’ self-ratings greatly overestimate their leadership performances relative to others’ ratings (Grijalva & Zhang, 2016; Rosenthal & Pittinsky, 2006). In addition, this finding offers valuable insights into the relationship between self-monitoring and self-other rating agreement (SOA) on leadership, which is defined as “agreement between self and other leadership ratings” (Atwater & Yammarino, 1992, p. 141). SOA is pivotal for leader performance (Fleenor et al., 2010). Inflated self-rating is problematic because overconfidence in leadership would lead to ineffective decision-making and refusal of necessary training and development (Bass & Yammarino, 1991; Lee & Carpenter, 2018). While Fleenor et al. (2010) posit that HSMs might achieve better SOA due to their attunement to interpersonal cues, our study finds the contrary, suggesting that leaders with higher levels of self-monitoring overestimate their leadership effectiveness.

Further, our findings offer valuable insights into the consequences of being an “emergent leader,” namely whether emergent leaders become “effective leaders” (Badura et al., 2022; Lanaj & Hollenbeck, 2015). The results suggest that HSMs are more likely to emerge as leaders than LSMs, but not necessarily more effective leaders, except in their own perception (self-rating). Therefore, HSMs are more likely to become *over-emergers*, that is, “instances when the level of one’s leadership emergence is higher than the level of one’s leadership effectiveness” (Lanaj & Hollenbeck, 2015, p. 1476). This prompts us to consider the implications of HSMs’ leadership emergence.

### Self-Monitoring

Our findings underscore the importance of examining self-monitoring in its dimensions and taking into account the measurement scales employed. The difference between RSMS and SMS-R in the relationship between self-monitoring and authentic leadership highlights the difference between self-monitoring motivation and ability. We found that while self-monitoring motivation (captured by SMS-R) undermines authentic leadership, self-monitoring abilities (captured by RSMS) are positively associated with it. By delving into self-monitoring motivation and abilities to comprehend their associations with authentic leadership, this study responds to Gardner et al.’s call (2011, p. 1138) to adopt “a more sophisticated understanding of the self-monitoring construct” to explore the complex relationship between self-monitoring and authentic leadership.

In addition, the results highlight differences between acquisitive motivation and protective motivation of self-monitoring. The relationship between self-monitoring and leadership emergence is significantly stronger when self-monitoring is measured by SMS-R than by SMS, indicating the acquisitive motive rather than the protective motive contributes to leadership emergence. This is because the shorter 18-item SMS-R omits seven items from the 25-item SMS, with the removed items mainly addressing the protective concern of self-monitoring (Briggs & Cheek, 1988). Consequently, the SMS-R emphasizes more on the acquisitive aspect of self-monitoring motivation, while the SMS comparatively focuses on the protective aspect of self-monitoring motivation. In addition, the relationships between self-monitoring and leadership effectiveness are significantly stronger for both RSMS and SMS-R than for SMS. Likewise, the relationship between self-monitoring and managerial leadership is stronger when the former is measured by RSMS than SMS. These results overall indicate that the protective motive has little impact on leadership.

### Leadership

This meta-analysis contributes to leadership research by extending the understanding of the leader trait paradigm (c.f., Judge et al., 2009) by providing a detailed quantitative analysis of the relationship between the self-monitoring trait and leadership. Judge et al. (2009) conceptually mapped the association between 12 traits and leadership emergence and effectiveness, naming it the Leader Trait Emergence Effectiveness (LTEE) heuristic model. Yet, they left out self-monitoring. Since “getting-along” and “getting-ahead” are important mediators in their model, based on socioanalytic theory (Hogan et al., 1985), self-monitoring should be a crucial trait within the framework because it concerns both motivations (Day & Schleicher, 2006). Thus, our findings contribute to a more comprehensive understanding of leadership from the leader trait perspective (Judge et al., 2009; Kaiser et al., 2008).

Our findings also shed light on the intricacies of the 360-degree leadership ratings (“full circle” assessment ratings from different sources, including supervisors, peers, subordinates, and even suppliers and customers, London & Smither, 1995) and SOA (Atwater & Yammarino, 1992) on leadership. Our results demonstrate that a leader’s motivational factors may contribute to variations in these ratings. For instance, when in the presence of subordinates, leaders with instrumental motives may pay less attention to their interpersonal behaviors. Understanding the reasons for leadership rating discrepancy is critical for gaining a deeper knowledge of leadership, as well as for informing leadership development (Atwater & Waldman, 1998).

Finally, our research offers valuable insights into the distinctiveness of leadership measures. Previous meta-analyses have suggested a potential redundancy in leadership variables.^4^ For instance, authentic leadership and transformational leadership have been subject to the critique of construct redundancy (Banks et al., 2016; Hoch et al., 2018). Our results indicate that their correlations with self-monitoring present a meaningful difference: a main effect size of .248 for authentic leadership compared to .094 for transformational leadership. This suggests that a possible distinction between these two leadership constructs lies in individual differences in self-monitoring, such that self-monitoring seems to be crucial for perceptions of authentic leadership. This finding aligns with the notion of “embodied authentic leadership” (Ladkin & Taylor, 2010), which suggests that authentic leaders should deliberately embody their “true selves” rather than freely expressing all emotions. Self-monitoring abilities may facilitate this by enabling leaders to make more informed choices about emotional expression and adapt appropriately when enacting leadership.

### Limitations and Future Research

One limitation of this meta-analysis is that it only examined linear correlations. Since meta-analyses draw on correlations, they only test linear relationships. This assumes a linear relationship between self-monitoring and the leadership-dependent variables. However, it is possible that there are nonlinear associations, which necessitates further research for investigation. For example, self-monitoring might show a nonlinear correlation with transformational leadership: when self-monitoring is very low, it hinders leaders’ capacity for individualized consideration and idealized influence; but when self-monitoring is very high, the chameleon aspect of self-monitoring casts doubt on leaders’ authenticity and principle, which negatively affects transformational leadership (Bass et al., 2003).

Another limitation of this meta-analysis is that some correlations are computed with a small number of studies, such as authentic leadership and transformational/transactional leadership. As a result, the interpretation of these results should be approached with caution. Since this study encompasses all available findings in the literature, it indicates that there is less research on the relationship between self-monitoring and these leadership constructs. Hence, future research could further examine their associations. In particular, future research could examine the relationship between self-monitoring and the components of these leadership variables since self-monitoring can have different impacts on the components. Further, no studies have explored the relationship between self-monitoring and other leadership styles, such as ethical leadership, servant leadership, parental leadership, or negative leadership styles like destructive leadership, representing a promising area for investigation in future studies.

With regard to the self-monitoring construct, although we have investigated self-monitoring motivation and ability by comparing different self-monitoring scales, there could be opportunities to conduct a finer-grained analysis of the two dimensions using scales that are more explicitly designed to separately capture these two dimensions (e.g., Warech et al., 1998). Operationalizing self-monitoring as separate sub-scales to capture each of its dimensions would greatly enhance our understanding of both self-monitoring and its relationship with leadership. For example, with separate measurements of the ability and motivation dimensions, there is potential to examine different types of LSMs, including those low in motivation or ability to self-monitor, or both, and their associations with leadership.

Understanding the dimensions of motivation and ability in self-monitoring will also enhance the differentiation between the self-monitoring construct and related concepts, such as social-monitoring — which focuses on attending to social cues and information (Gardner, Pickett, et al., 2005) — and Machiavellianism — which is characterized by manipulativeness and the drive to use any means necessary for self-gain (Lange et al., 2018). The sensitivity aspect of self-monitoring ability aligns with the concept of social-monitoring. However, while social-monitoring seeks social acceptance and belongingness (Gardner, Pickett, et al., 2005), which relates to the “getting along” motivation of self-monitoring, it doesn’t align with the “getting ahead” motivation of self-monitoring, which aims for elevated status among others. Regarding Machiavellianism, this dark triad trait aligns with self-monitoring in its acquisitive motivation for power and status attainment (Hawley, 2003), driven by self-centered interest (Jones & Paulhus, 2017). This shared motivation underscores the positive correlation between self-monitoring and Machiavellianism (Kowalski et al., 2018). However, unlike Machiavellianism, self-monitoring is not inherently manipulative and also incorporates the abilities to adapt to social appropriateness. Future research could examine the nuanced associations of these constructs by considering the self-monitoring dimensions.

### Practical Implications

Previous research (e.g., Day et al., 2002) indicates a positive relationship between self-monitoring and leadership. However, my findings reveal a more nuanced picture, indicating that the positive relationship should be interpreted with caution from a practical standpoint. Self-monitoring generally shows a non-significant relationship with subordinate-rated leadership. This implies that practitioners should be cautious not to be easily impressed by individuals skilled at cultivating favorable social images. The ability to meet situational demands does not necessarily translate to effective leadership. On the contrary, individuals who may not seem adept at adapting to situations (but showcasing strong principles) might still possess valuable leadership qualities and should be given equal opportunities in leadership roles.

Practitioners are advised to closely examine those who emerge as leaders to ensure that they are not only successful in the process of emergence but also effective leaders in practice. Organizations can implement 360-degree appraisals for their managers and leaders, gathering ratings from multiple sources (supervisor, peer, subordinate, and self) to gain a comprehensive and more accurate assessment, especially when dealing with managers or leaders who exhibit high self-monitoring tendencies.

Furthermore, the findings suggest that self-monitoring ability contributes positively to leadership, particularly authentic leadership, while self-monitoring motivation might undermine it. Leaders are encouraged to nurture their abilities in sensitivity and self-presentation abilities. However, they should be cautious not to use these abilities for chameleon-like behaviors and opportunistic gains. Instead, leaders should use these abilities as valuable resources with authenticity, that is, with a lower level of self-monitoring motivation. Therefore, leaders can be both socially capable and principled, aligning with what Bedeian (Bedeian & Day, 2004) referred to as “real” leaders.

## Supplemental Material

sj-docx-1-psp-10.1177_01461672231210778 – Supplemental material for Do Chameleons Lead Better?: A Meta-Analysis of the Self-Monitoring and Leadership Relationship Supplemental material, sj-docx-1-psp-10.1177_01461672231210778 for Do Chameleons Lead Better?: A Meta-Analysis of the Self-Monitoring and Leadership Relationship by Linghe Lei, Chen Wang and Jonathan Pinto in Personality and Social Psychology Bulletin

sj-docx-2-psp-10.1177_01461672231210778 – Supplemental material for Do Chameleons Lead Better?: A Meta-Analysis of the Self-Monitoring and Leadership Relationship Supplemental material, sj-docx-2-psp-10.1177_01461672231210778 for Do Chameleons Lead Better?: A Meta-Analysis of the Self-Monitoring and Leadership Relationship by Linghe Lei, Chen Wang and Jonathan Pinto in Personality and Social Psychology Bulletin

sj-docx-3-psp-10.1177_01461672231210778 – Supplemental material for Do Chameleons Lead Better?: A Meta-Analysis of the Self-Monitoring and Leadership Relationship Supplemental material, sj-docx-3-psp-10.1177_01461672231210778 for Do Chameleons Lead Better?: A Meta-Analysis of the Self-Monitoring and Leadership Relationship by Linghe Lei, Chen Wang and Jonathan Pinto in Personality and Social Psychology Bulletin

sj-docx-4-psp-10.1177_01461672231210778 – Supplemental material for Do Chameleons Lead Better?: A Meta-Analysis of the Self-Monitoring and Leadership Relationship Supplemental material, sj-docx-4-psp-10.1177_01461672231210778 for Do Chameleons Lead Better?: A Meta-Analysis of the Self-Monitoring and Leadership Relationship by Linghe Lei, Chen Wang and Jonathan Pinto in Personality and Social Psychology Bulletin

## Notes

1. Leadership emergence, which refers to perceptions of leadership attributed to an individual not holding a formal leader role, is typically not assessed by subordinates (Hanna et al., 2021). However, in our sample, subordinates were involved in providing leadership perception ratings for the focal leader (e.g., D. R. Campbell, 1993), using the same measures as for leadership emergence. This enabled us to compare subordinate ratings with other evaluations, thus providing further insights.

2. SMS was not compared because there were no data for this analysis.

3. Repository: https://osf.io/fd2q6/

4. We provided correlations between leadership variables included in our meta-analysis, as retrieved from previous meta-analytic studies. These can be found in Online Supplement C.
