# Supplementary material for: Do Chameleons Lead Better? A Meta-Analysis of the Self-Monitoring and Leadership Relationship
Source: Pers Soc Psychol Bull. 2023 Nov 25;51(7):1139–58. doi: 10.1177/01461672231210778 (PMC12130612; doi:10.1177/01461672231210778)
Supplement: sj-docx-1-psp-10.1177_01461672231210778 – Supplemental material for Do Chameleons Lead Better?: A Meta-Analysis of the Self-Monitoring and Leadership Relationship [file sj-docx-1-psp-10.1177_01461672231210778.docx]

**Online Supplement A. Results with Egger’s Test**

**Table 1**

*Self-monitoring and Leadership: Main Effects*

| Leadership Variables | | Original Effects | | | | |  | Egger's test | |  |  | Trim and Fill |  |
| --- | --- | --- | --- | --- | --- | --- | --- | --- | --- | --- | --- | --- | --- |
|  |  | *k* | *N* | *ρ* | SD*_ρ_* | 95% CI |  | *z*-value | *p*-value |  | TF *ρ* | 95% CI | Missing# |
| Leadership Emergence | | 25 | 3355 | **.171** | .031 | [.110, .232] |  | 0.818 | .413 |  | .164 | [.100, .229] | 1 |
| Leadership Effectiveness | | 25 | 3162 | **.123** | .032 | [.061, .184] |  | **-2.198** | .028 |  | .182 | [.108, .257] | 8 |
| Authentic Leadership | | 5 | 594 | **.248** | .100 | [.053, .444] |  | -1.578 | .115 |  | .334 | [.106, .561] | 2 |
| Transformational Leadership | | 7 | 829 | **.094** | .043 | [.010, .179] |  | 0.429 | .668 |  | .094 | [.009, .179] | 0 |
| Transactional Leadership | | 3 | 395 | -.005 | .074 | [-.150, .141] |  | 1.881 | .060 |  | -.005 | [-.150, .141] | 0 |
| Managerial Leadership | | 18 | 3802 | **.121** | .035 | [.053, .190] |  | 1.863 | .062 |  | .087 | [-.008, .181] | 6 |
|  | Task-oriented Behavior | 12 | 1264 | **.205** | .039 | [.129, .281] |  | 0.264 | .792 |  | .205 | [.129, .281] | 0 |
|  | Relationship-oriented Behavior | 13 | 1308 | **.100** | .042 | [.018, .182] |  | -0.423 | .672 |  | .111 | [.024, .198] | 1 |

*Note.* Effects in **bold** are statistically significant.

*k* = number of samples; *N* = total sample size; *r* = sample-size weighted average correlation; SD_r_ = standard deviation of sample-size weighted average correlation; *ρ* = estimated true-score correlation; SD*_ρ_* = standard deviation of estimated true-score correlation; 95% CI = 95% confidence interval; Missing# = estimated No. of missing studies; Managerial Leadership includes generic leadership behaviors not categorized as either task/relationship-oriented behavior by the original author(s)

**Table 2**

*Moderation Effects of Self-monitoring Measurement Scale*

| Leadership Variables | Original Effects | | | | |  | Egger's test | |  | Trim and Fill | | |
| --- | --- | --- | --- | --- | --- | --- | --- | --- | --- | --- | --- | --- |
|  | *k* | *N* | *ρ* | SD*_ρ_* | 95% CI |  | *z*-value | *p*-value |  | TF *ρ* | 95% CI | Missing# |
| Leadership Emergence |  |  |  |  |  |  |  |  |  |  |  |  |
| RSMS | 17 | 2421 | **.175** | .038 | [.099, .250] |  | 0.555 | .579 |  | .175 | [.099, .250] | 0 |
| SMS | 5 | 705 | .086 | .047 | [-.006, .178] |  | 1.705 | .088 |  | .060 | [-.031, .151] | 1 |
| SMS-R | 5 | 517 | **.266** | .052 | [.165, .367] |  | 0.039 | .969 |  | .266 | [.165, .367] | 0 |
| Leadership Effectiveness |  |  |  |  |  |  |  |  |  |  |  |  |
| RSMS | 9 | 1638 | **.175** | .036 | [.104, .246] |  | -1.475 | .140 |  | .205 | [.125, .285] | 3 |
| SMS | 9 | 669 | -.036 | .059 | [-.152, .080] |  | 0.638 | .524 |  | -.050 | [-.172, .073] | 1 |
| SMS-R | 7 | 855 | **.131** | .042 | [.049, .213] |  | 0.086 | .931 |  | .131 | [.049, .213] | 0 |
| Authentic Leadership |  |  |  |  |  |  |  |  |  |  |  |  |
| RSMS | 2 | 370 | **.391** | .061 | [.270, .511] |  |  |  |  |  |  |  |
| SMS-R | 2 | 166 | .048 | .109 | [-.165, .261] |  |  |  |  |  |  |  |
| Transformational Leadership | |  |  |  |  |  |  |  |  |  |  |  |
| SMS | 3 | 210 | .073 | .092 | [-.108, .254] |  | 0.985 | .325 |  | -.026 | [-.170, .117] | 2 |
| SMS-R | 3 | 471 | **.159** | .057 | [.048, .270] |  | 0.615 | .538 |  | .143 | [.039, .247] | 1 |
| Managerial Leadership |  |  |  |  |  |  |  |  |  |  |  |  |
| RSMS | 8 | 1061 | **.215** | .039 | [.138, .292] |  | -0.023 | .981 |  | .215 | [.138, .292] | 0 |
| SMS | 6 | 2207 | **.067** | .028 | [.013, .121] |  | 0.358 | .721 |  | .064 | [.010, .117] | 2 |
| SMS-R | 6 | 822 | **.127** | .043 | [.042, .212] |  | 1.873 | .061 |  | .104 | [.002, .205] | 1 |

*Note.* Effects in **bold** are statistically significant.

*k* = number of samples; *N* = total sample size; *r* = sample-size weighted average correlation; SD_r_ = standard deviation of sample-size weighted average correlation; *ρ* = estimated true-score correlation; SD*_ρ_* = standard deviation of estimated true-score correlation; 95% CI = 95% confidence interval; Missing# = estimated No. of missing studies.

We only conducted analysis when at least 2 studies reported the correlation, and some subcategory analysis cannot be implemented. The addition of subcategory *k* can be larger than the total *k* because some studies include more than one subcategory datapoint.

**Table 3**

*Moderation Effects of Leadership Rating Source*

| Leadership Variables | Original Effects | | | | |  | Egger's test | |  | Trim and Fill | | |
| --- | --- | --- | --- | --- | --- | --- | --- | --- | --- | --- | --- | --- |
|  | *k* | *N* | *ρ* | SD*_ρ_* | 95% CI |  | *z*-value | *p*-value |  | TF *ρ* | 95% CI | Missing# |
| Leadership Emergence | |  |  |  |  |  |  |  |  |  |  |  |
| Subordinate | 2 | 174 | .135 | .102 | [-.064, .334] |  |  |  |  |  |  |  |
| Peer | 19 | 2568 | **.158** | .038 | [.084, .232] |  | 0.976 | .329 |  | .149 | [.070, .227] | 1 |
| Observer | 3 | 425 | **.178** | .068 | [.045, .312] |  | -1.946 | .052 |  | .244 | [.075, .412] | 2 |
| Self | 3 | 259 | **.269** | .095 | [.082, .456] |  | -1.701 | .089 |  | .269 | [.082, .456] | 0 |
| Leadership Effectiveness | |  |  |  |  |  |  |  |  |  |  |  |
| Subordinate | 9 | 1075 | .034 | .049 | [-.063, .131] |  | -1.312 | .190 |  | .091 | [-.027, .210] | 2 |
| Self | 5 | 1222 | **.223** | .051 | [.124, .323] |  | 0.230 | .818 |  | .223 | [.124, .323] | 0 |
| Supervisor | 13 | 1323 | .071 | .046 | [-.019, .160] |  | -1.765 | .078 |  | .071 | [-.019, .160] | 0 |
| Authentic Leadership |  |  |  |  |  |  |  |  |  |  |  |  |
| Subordinate | 3 | 455 | **.261** | .098 | [.070, .452] |  | **-2.745** | .006 |  | .373 | [.145, .600] | 2 |
| Self | 3 | 197 | .138 | .150 | [-.155, .432] |  | -0.550 | .583 |  | .138 | [-.155, .432] | 0 |
| Transformational Leadership | |  |  |  |  |  |  |  |  |  |  |  |
| Subordinate | 7 | 829 | .064 | .044 | [-.021, .150] |  | 1.071 | .284 |  | .037 | [-.043, .117] | 2 |
| Transactional Leadership | |  |  |  |  |  |  |  |  |  |  |  |
| Subordinate | 3 | 395 | -.005 | .074 | [-.150, .141] |  | 1.881 | .060 |  | -.005 | [-.150, .141] | 0 |
| Managerial Leadership | |  |  |  |  |  |  |  |  |  |  |  |
| Subordinate | 8 | 675 | .058 | .075 | [-.089, .204] |  | 1.442 | .149 |  | .006 | [-.150, .162] | 3 |
| Peer | 4 | 463 | **.272** | .059 | [.157, .386] |  | -0.010 | .992 |  | .272 | [.157, .386] | 0 |
| Observer | 5 | 2481 | **.093** | .040 | [.015, .172] |  | 1.000 | .317 |  | .093 | [.015, .172] | 0 |
| Self | 8 | 748 | .041 | .053 | [-.064, .145] |  | 0.094 | .925 |  | .041 | [-.064, .145] | 0 |
| Supervisor | 3 | 402 | **.184** | .062 | [.063, .305] |  | -0.487 | .626 |  | .208 | [.108, .307] | 2 |

*Note.* Effects in **bold** are statistically significant.

*k* = number of samples; *N* = total sample size; *r* = sample-size weighted average correlation; SD_r_ = standard deviation of sample-size weighted average correlation; *ρ* = estimated true-score correlation; SD*_ρ_* = standard deviation of estimated true-score correlation; 95% CI = 95% confidence interval; Missing# = estimated No. of missing studies.

We only conducted analysis when at least 2 studies reported the correlation, and some subcategory analysis cannot be implemented. The addition of subcategory *k* can be larger than the total *k* because some studies include more than one subcategory datapoint. The rating source indicates rating by this source only, excluding those with multiple rating sources.
