# Supplementary material for: Do Chameleons Lead Better? A Meta-Analysis of the Self-Monitoring and Leadership Relationship
Source: Pers Soc Psychol Bull. 2023 Nov 25;51(7):1139–58. doi: 10.1177/01461672231210778 (PMC12130612; doi:10.1177/01461672231210778)

**Online Supplement B. Funnel Plot (Main Effect)**

**Figure 1**

*Funnel Plot for Self-monitoring and Leadership Emergence*

**
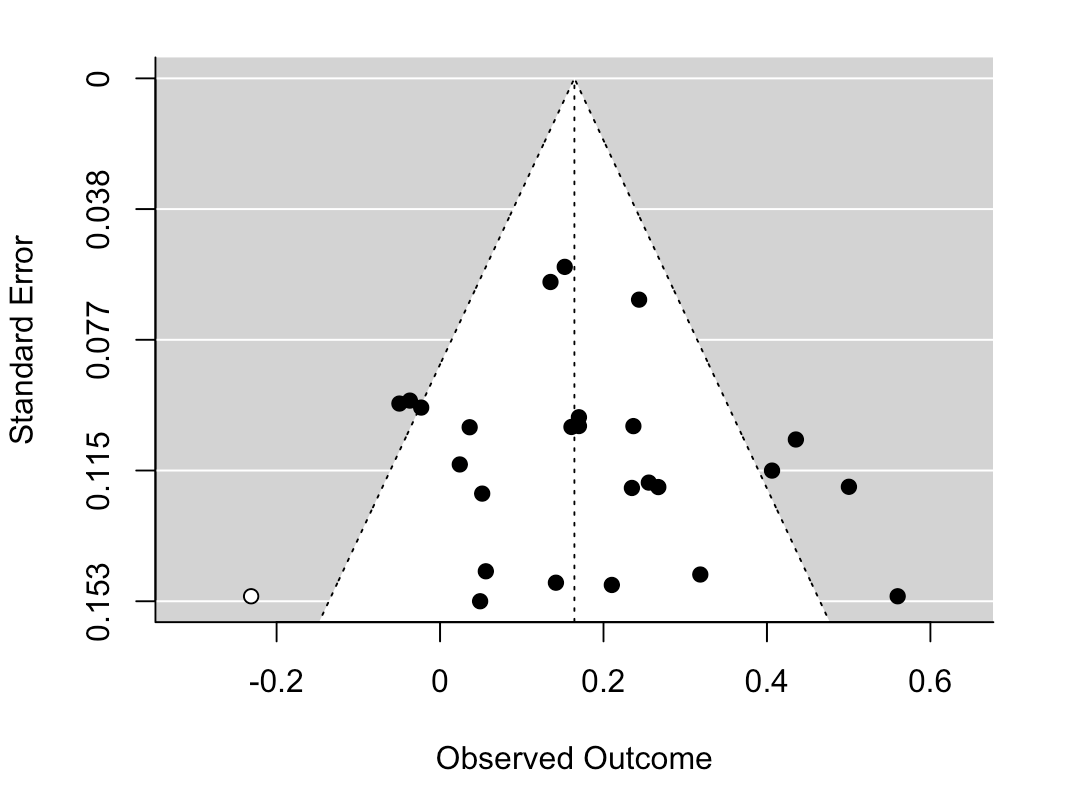
**

**Figure 2**

*Funnel Plot for Self-monitoring and Leadership Effectiveness*


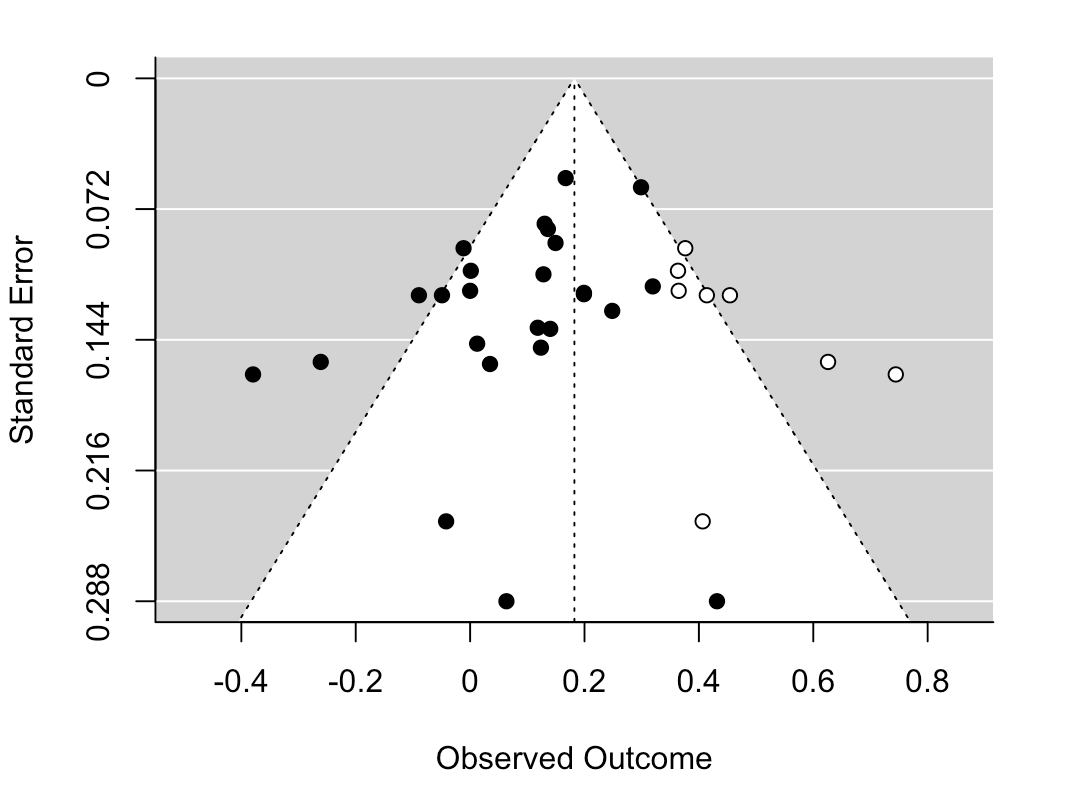


**Figure 3**

*Funnel Plot for Self-monitoring and Authentic Leadership*

**
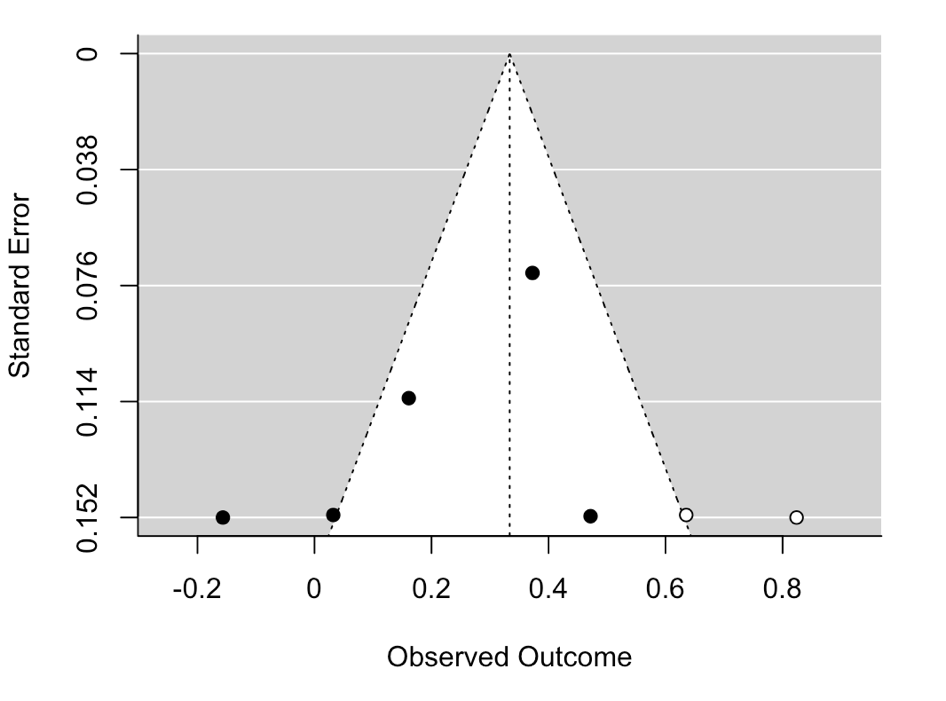
**

**Figure 4**

*Funnel Plot for Self-monitoring and Transformational Leadership*


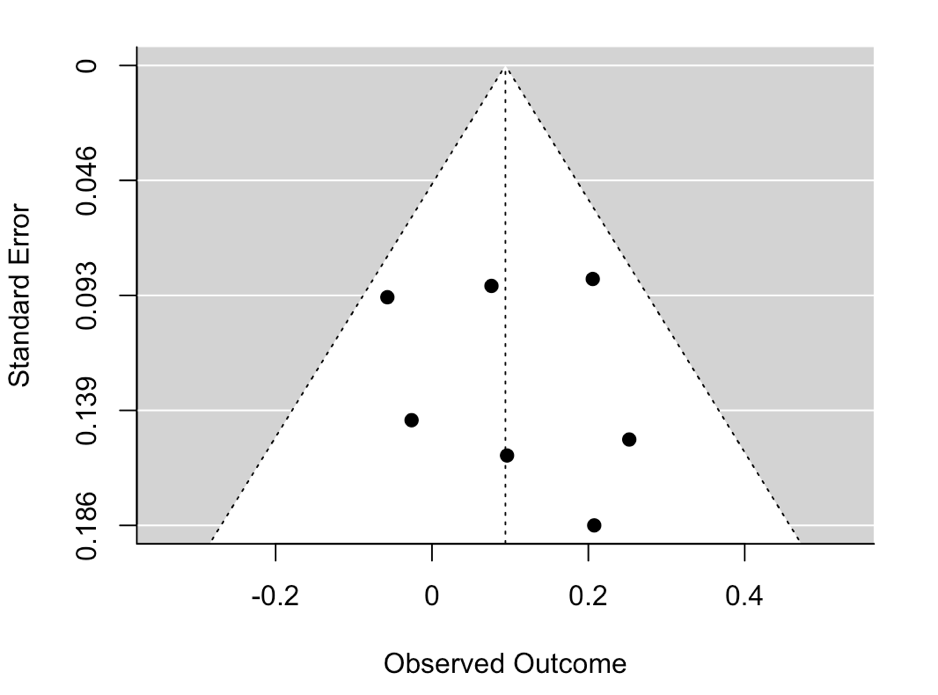


**Figure 5**

*Funnel Plot for* *Self-monitoring and Transactional Leadership*


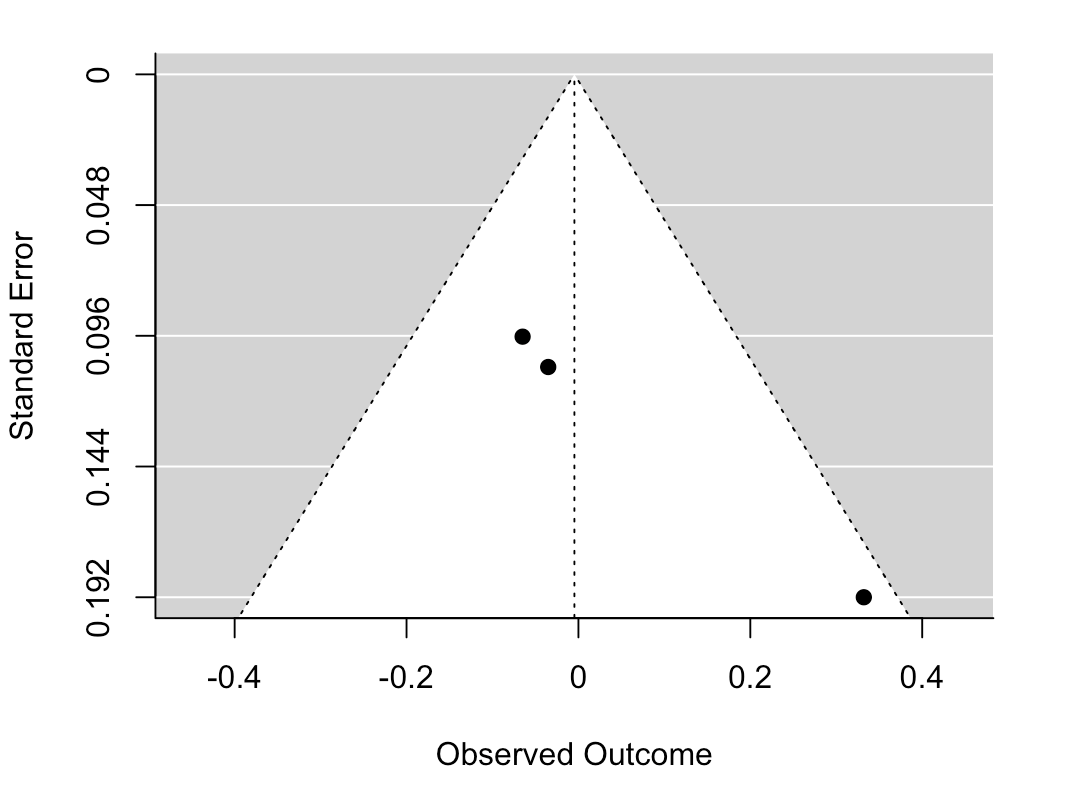


**Figure 6**

*Funnel Plot for Self-monitoring and Managerial Leadership*


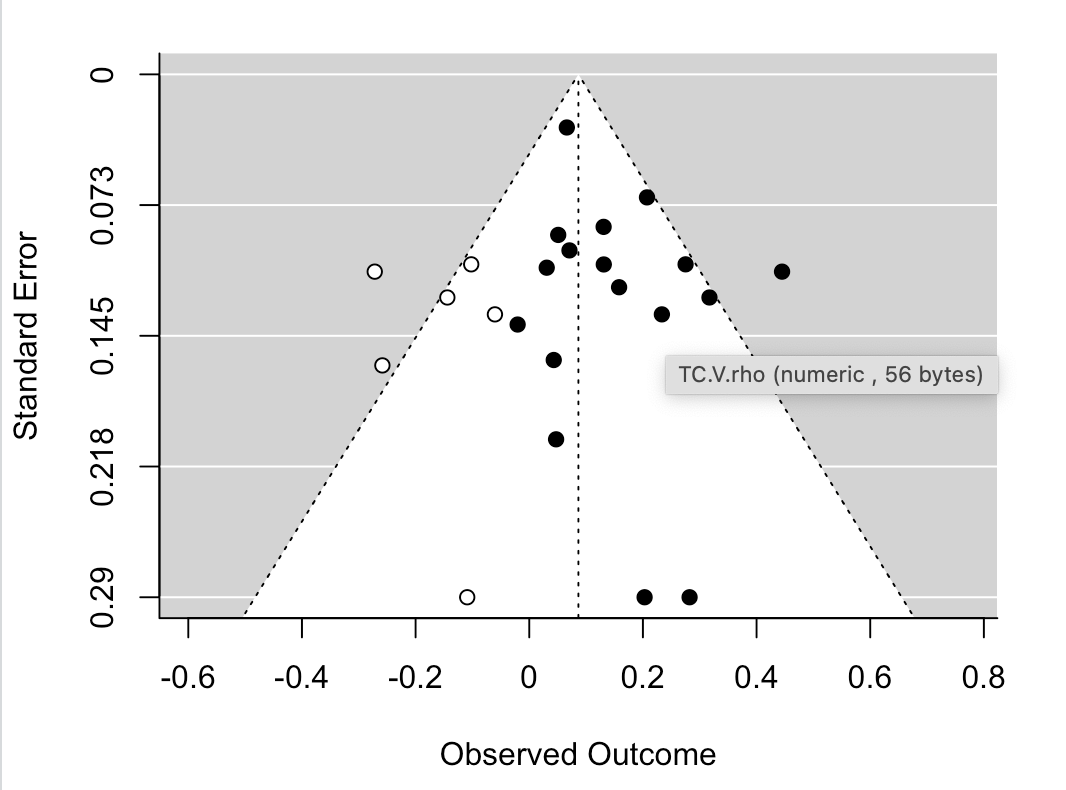

Supplement: sj-docx-2-psp-10.1177_01461672231210778 – Supplemental material for Do Chameleons Lead Better?: A Meta-Analysis of the Self-Monitoring and Leadership Relationship [file sj-docx-2-psp-10.1177_01461672231210778.docx]
