# Supplementary material for: Do Chameleons Lead Better? A Meta-Analysis of the Self-Monitoring and Leadership Relationship
Source: Pers Soc Psychol Bull. 2023 Nov 25;51(7):1139–58. doi: 10.1177/01461672231210778 (PMC12130612; doi:10.1177/01461672231210778)
Supplement: sj-docx-3-psp-10.1177_01461672231210778 – Supplemental material for Do Chameleons Lead Better?: A Meta-Analysis of the Self-Monitoring and Leadership Relationship [file sj-docx-3-psp-10.1177_01461672231210778.docx]

**Online Supplement C. Correlations between Leadership Variables**

**Table 1**

*Intercorrelations among variables leadership emergence, leadership effectiveness, authentic leadership, transformational leadership, transactional leadership, and managerial leadership*

|  |  | 1 | 2 | 3 | 4 | 5 | 6 |
| --- | --- | --- | --- | --- | --- | --- | --- |
| 1 | Leadership emergence | - |  |  |  |  |  |
| 2 | Leadership effectiveness | n.r. | - |  |  |  |  |
| 3 | Authentic leadership | n.r. | *ρ=*.69, *k*=13, *N*=3,812, [.53, .84] ^a^ | - |  |  |  |
| 4 | Transformational leadership | n.r. | *ρ=*.39, *k*=100, *N*=13221, [.33, .44] ^b^ | *ρ=*.75, *k*=10, *N*=2397, [.58, .92] ^c^ | - |  |  |
| 5 | Transactional leadership | n.r. | *ρ=*.12, *k*=26, *N*=2,838, [.01, .23] ^b^ | *ρ=*.40, *k*=4, *N*=770, [.03, .86] ^c^ | Contingent reward *ρ=*.80, *k*=87, *N*=22,369, [n.r.]; MBE-active *ρ=*.17, *k*=60, *N*=12,600, [n.r.]; MBE-passive *ρ=* -.20, *k*=50, *N*=10,928, [n.r.] ^d^ | - |  |
| 6 | Managerial leadership | n.r. | Initiating structure *ρ=*.39, *k*=20, *N*=1,960, [n.r.]; Consideration *ρ=*.52, *k*=20, *N*=1,605, [n.r.] ^e^ | n.r. | n.r. | n.r. | - |

*Note.* Coefficients presented in the table represent correlations corrected for unreliability (*ρ)*

*k* = number of independent effect sizes; *N* = total sample size; values in [ ] represent 95% confidence interval for *ρ*; n.r.= not reported; MBE=Management by exception

^a^ Retrieved from Zhang et al. (2022)

^b^ Retrieved from Rockstuhl et al. (2023)

^c^ Retrieved from Hoch et al. (2018)

^d^ Retrieved from Judge & Piccolo (2004)

^e^ Retrieved from Judge et al. (2004)

**References**

Hoch, J. E., Bommer, W. H., Dulebohn, J. H., & Wu, D. (2018). Do ethical, authentic, and servant leadership explain variance above and beyond transformational leadership? A meta-analysis. *Journal of Management, 44*(2), 501-529.

Judge, T. A., & Piccolo, R. F. (2004). Transformational and transactional leadership: a meta-analytic test of their relative validity. *Journal of Applied Psychology, 89*(5), 755-768.

Judge, T. A., Piccolo, R. F., & Ilies, R. (2004). The forgotten ones? The validity of consideration and initiating structure in leadership research. *Journal of Applied Psychology, 89*(1), 36-51.

Rockstuhl, T., Wu, D., Dulebohn, J. H., Liao, C., & Hoch, J. E. (2023). Cultural congruence or compensation? A meta-analytic test of transformational and transactional leadership effects across cultures. *Journal of International Business Studies, 54*(3), 476-504.

Zhang, Y., Guo, Y., Zhang, M., Xu, S., Liu, X., & Newman, A. (2022). Antecedents and outcomes of authentic leadership across culture: A meta-analytic review. *Asia Pacific Journal of Management, 39*(4), 1399-1435.
