# Supplementary material for: Do Chameleons Lead Better? A Meta-Analysis of the Self-Monitoring and Leadership Relationship
Source: Pers Soc Psychol Bull. 2023 Nov 25;51(7):1139–58. doi: 10.1177/01461672231210778 (PMC12130612; doi:10.1177/01461672231210778)
Supplement: sj-docx-4-psp-10.1177_01461672231210778 – Supplemental material for Do Chameleons Lead Better?: A Meta-Analysis of the Self-Monitoring and Leadership Relationship [file sj-docx-4-psp-10.1177_01461672231210778.docx]

**Online Supplement D. List of Studies Included in the Meta-Analysis**

Anderson, L. R., & McLenigan, M. (1987). Sex differences in the relationship between self-monitoring and leader behavior. *Small Group Behavior, 18*(2), 147-167.

Bastaman, A. M., Riantoputra, C. D., & Gatari, E. (2018). Do self-monitoring and achievement orientation assist or limit leader effectiveness? *Diversity in Unity: Perspectives from Psychology and Behavioral Sciences* (p. 355-364). Routledge/Taylor & Francis Group.

Becker, J. T. (2003). *Factors contributing to differences in multisource performance ratings: The role of self-monitoring, rating source and dimension.* Unpublished doctoral dissertation, Illinois Institute of Technology.

Buckner V, J. E. (2012). *Emotional labor and authentic leadership*. Unpublished doctoral dissertation, Louisiana Tech University

Campbell, D. R. (1993). *An investigation of the relationship between self-monitoring and leader effectiveness*. Unpublished doctoral dissertation, The University of Tennessee.

Chaudhary, R., & Panda, C. (2019). Examining self-monitoring and neuroticism as predictors and self-efficacy as an outcome of Authentic leadership. *Organization Management Journal, 16*(3), 179-192.

Church, A. H. (1997). Managerial self-awareness in high-performing individuals in organizations. *Journal of Applied Psychology*, *82*(2), 281-292.

Côté, S., Lopes, P. N., Salovey, P., & Miners, C. T. (2010). Emotional intelligence and leadership emergence in small groups. *The Leadership Quarterly, 21*(3), 496-508.

Dobbins, G. H., Long, W. S., Dedrick, E. J., & Clemons, T. C. (1990). The role of self-monitoring and gender on leader emergence: A laboratory and field study. *Journal of Management*, *16*(3), 609-618.

Dobosh, M. A. (2005). *The impact of cognitive complexity and self-monitoring on leadership emergence.* Unpublished doctoral dissertation, University of Delaware.

Douglas, C., & Ammeter, A. P. (2004). An examination of leader political skill and its effect on ratings of leader effectiveness. *The Leadership Quarterly, 15*(4), 537-550.

Eby, L. T., Cader, J., & Noble, C. L. (2003). Why Do High Self‐Monitors Emerge as Leaders in Small Groups? A Comparative Analysis of the Behaviors of High Versus Low Self‐Monitors. *Journal of Applied Social Psychology*, *33*(7), 1457-1479.

Eisenberg, A. P. (1999). *The search for integrity: A leadership impact study.* Unpublished doctoral dissertation, DePaul University.

Ellis, R. J. (1988). Self-monitoring and leadership emergence in groups. *Personality and Social Psychology Bulletin, 14*(4), 681-693.

Foti, R. J., & Hauenstein, N. (2007). Pattern and variable approaches in leadership emergence and effectiveness. *Journal of Applied Psychology*, *92*(2), 347-355.

Garland, H., & Beard, J. F. (1979). Relationship between self-monitoring and leader emergence across two task situations. *Journal of Applied Psychology*, *64*(1), 72-76.

Haley, M. J. (1983). *Relationship between internal-external locus of control beliefs, self-monitoring and leadership style adaptability.* Unpublished doctoral dissertation, Case Western Reserve University.

Hall, R. J., Workman, J. W., & Marchioro, C. A. (1998). Sex, task, and behavioral flexibility effects on leadership perceptions. *Organizational Behavior and Human Decision Processes*, *74*(1), 1-32.

Hatch, D. D. (1987). *The relationship between self-monitoring and management performance in males and females in a field setting.* Unpublished doctoral dissertation, Brigham Young University.

Joplin, J. R. W. (1994). *Constructing and testing a model of leadership development processes in organizations.* Unpublished doctoral dissertation, The University of Texas at Arlington.

Jung, D. & Sosik, J. J. (2006). Who are the spellbinders? Identifying personal attributes of charismatic leaders. *Journal of Leadership & Organizational Studies*, *12*(4), 12-26.

Kent, R. L., & Moss, S. E. (1990). Self-monitoring as a predictor of leader emergence. *Psychological Reports*, *66*(3), 875-881.

Kilduff, M., & Day, D. V. (1994). Do chameleons get ahead? The effects of self-monitoring on managerial careers. *Academy of Management Journal*, 37(4), 1047-1060.

Kilduff, M., Mehra, A., Gioia, D. A. D., & Borgatti, S. (2017). Brokering trust to enhance leadership: A self-monitoring approach to leadership emergence. *In Knowledge and Networks* (pp. 221-240). Springer, Cham.

Law, J. R. (1996). *Rising to the occasion: Foundations, processes, and outcomes of emergent leadership.* Unpublished doctoral dissertation, The University of Texas at Austin.

Lewis, T. M. (2010). *The influence of authenticity and emotional intelligence on the relationship between self-monitoring and leadership effectiveness.* Unpublished doctoral dissertation, Alliant International University, Los Angeles.

Mehra, A. (1998). *Who gets ahead? Self-monitoring, social networks, and success in organizations.* Unpublished doctoral dissertation, The Pennsylvania State University.

Morath, R. A. (1999). *Leader abilities and attributes: Their influence on ratings of assessment center and job performance.* Unpublished doctoral dissertation, George Mason University.

Oddou, G. R. (1983). *The emergence of leaders in natural work groups: A test of self-monitoring theory.* Unpublished doctoral dissertation, Brigham Young University.

Rubin, R. S., Bartels, L. K., & Bommer, W. H. (2002). Are leaders smarter or do they just seem that way? Exploring perceived intellectual competence and leadership emergence. *Social Behavior and Personality: An International Journal, 30*(2), 105-118.

Rutti, R. M. (2009). Followers who lead: Modeling leader emergence through self-monitoring and social identity theories (Doctoral dissertation, University of South Carolina).

Semadar, A., Robins, G., & Ferris, G. R. (2006). Comparing the validity of multiple social effectiveness constructs in the prediction of managerial job performance. *Journal of Organizational Behavior*, *27*(4), 443-461.

Sendjaya, S., Pekerti, A., Härtel, C., Hirst, G., & Butarbutar, I. (2016). Are authentic leaders always moral? The role of Machiavellianism in the relationship between authentic leadership and morality. *Journal of Business Ethics*, *133*(1), 125-139.

Shivers, S. L. (1999). *A role theory approach to understanding transformational and transactional leadership behaviors: The role of interpretations of organizational context.* Unpublished doctoral dissertation, Purdue University.

Simmons, K. B. (1994). *Determinants of functional leadership and leader emergence in long-term decision-making groups: A role theory approach.* Unpublished doctoral dissertation, Northwestern University.

Sosik, J. J., Avolio, B. J., & Jung, D. I. (2002). Beneath the mask: Examining the relationship of self‐presentation attributes and impression management to charismatic leadership. *The Leadership Quarterly*, *13*, 217–242.

Sosik, J. J., & Dinger, S. L. (2007). Relationships between leadership style and vision content: The moderating role of need for social approval, self-monitoring, and need for social power. *The Leadership Quarterly*, *18*(2), 134-153.

Sosik, J. J., Jung, D., & Dinger, S. L. (2009). Values in authentic action: Examining the roots and rewards of altruistic leadership. *Group & Organization Management*, *34*(4), 395-431.

Sosik, J. J., & Megerian, L. E. (1999). Understanding leader emotional intelligence and performance: The role of self-other agreement on transformational leadership perceptions. *Group & Organization Management*, *24*(3), 367-390.

Sosik, J. J., Potosky, D., & Jung, D. I. (2002). Adaptive self-regulation: Meeting others' expectations of leadership and performance. *The Journal of Social Psychology*, *142*(2), 211-232.

Tate, B. (2008). A longitudinal study of the relationships among self-monitoring, authentic leadership, and perceptions of leadership. *Journal of Leadership & Organizational Studies*, *15*(1), 16-29.

Thomas, J. L. (1999). *Personality and motivational predictors of military leadership assessment in the United States Army Reserve Officer Training Corps.* Unpublished doctoral dissertation, Wayne State University.

Tiedemann, K. F. (2004). *Integrity in military service: A leadership impact study.* Unpublished doctoral dissertation, University of Phoenix.

Toegel, G., Anand, N., & Kilduff, M. (2007). Emotion helpers: The role of high positive affectivity and high self‐monitoring managers. *Personnel Psychology, 60*(2), 337-365.

Türetgen, I. O., Unsal, P., & Dural, U. (2017). The role of leadership perception as a mediator between managers’ self-monitoring and subordinate attitudes. *The Journal of Social Psychology*, *157*(3), 295-307.

Türetgen, I. Ö., Unsal, P., & Erdem, I. (2008). The effects of sex, gender role, and personality traits on leader emergence: Does culture make a difference?. *Small Group Research*, *39*(5), 588-615.

Van Der Heide, B. L. (2006). *Why do high self-monitors emerge as group leaders?.* Unpublished doctoral dissertation, Michigan State University.

Verdigets, F. L. (2008). *Insights into leadership: The role of political skill, social skill, and self-monitoring in mediating the relationship between transformational leadership and job performance.* Unpublished doctoral dissertation, Auburn University.

Walsh, M. C. (1995). *The predictive value of psychological type and self-monitoring on leadership and leadership perceptions.* Unpublished doctoral dissertation, Virginia Tech.

Walter, F., Cole, M. S., van der Vegt, G. S., Rubin, R. S., & Bommer, W. H. (2012). Emotion recognition and emergent leadership: Unraveling mediating mechanisms and boundary conditions. *The Leadership Quarterly, 23*(5), 977-991.

Zaccaro, S. J., Foti, R. J., & Kenny, D. A. (1991). Self-monitoring and trait-based variance in leadership: An investigation of leader flexibility across multiple group situations. *Journal of Applied Psychology*, *76*(2), 308-315.
